# The MMP-2 histone H3 N-terminal tail protease is selectively targeted to the transcription start sites of active genes

**DOI:** 10.1186/s13072-023-00491-w

**Published:** 2023-05-10

**Authors:** Benjamin H. Weekley, Judd C. Rice

**Affiliations:** Department of Biochemistry and Molecular Medicine, University of Southern California Keck School of Medicine, 1450 Biggy Street, HNRT 6506, Los Angeles, CA 90033 USA

**Keywords:** Chromatin, Histone H3, MMP-2, CTSB, U2OS, Proteolysis

## Abstract

**Background:**

Proteolysis of the histone H3 N-terminal tail (H3NT) is an evolutionarily conserved epigenomic feature of nearly all eukaryotes, generating a cleaved H3 product that is retained in ~ 5–10% of the genome. Although H3NT proteolysis within chromatin was first reported over 60 years ago, the genomic sites targeted for H3NT proteolysis and the impact of this histone modification on chromatin structure and function remain largely unknown. The goal of this study was to identify the specific regions targeted for H3NT proteolysis and investigate the consequence of H3NT “clipping” on local histone post-translational modification (PTM) dynamics.

**Results:**

Leveraging recent findings that matrix metalloproteinase 2 (MMP-2) functions as the principal nuclear H3NT protease in the human U2OS osteosarcoma cell line, a ChIP-Seq approach was used to map MMP-2 localization genome wide. The results indicate that MMP-2 is selectively targeted to the transcription start sites (TSSs) of protein coding genes, primarily at the + 1 nucleosome. MMP-2 localization was exclusive to highly expressed genes, further supporting a functional role for H3NT proteolysis in transcriptional regulation. MMP-2 dependent H3NT proteolysis at the TSSs of these genes resulted in a > twofold reduction of activation-associated histone H3 PTMs, including H3K4me3, H3K9ac and H3K18ac. One of genes requiring MMP-2 mediated H3NT proteolysis for proficient expression was the lysosomal cathepsin B protease (CTSB), which we discovered functions as a secondary nuclear H3NT protease in U2OS cells.

**Conclusions:**

This study revealed that the MMP-2 H3NT protease is selectively targeted to the TSSs of active protein coding genes in U2OS cells. The resulting H3NT proteolysis directly alters local histone H3 PTM patterns at TSSs, which likely functions to regulate transcription. MMP-2 mediated H3NT proteolysis directly activates CTSB, a secondary H3NT protease that generates additional cleaved H3 products within chromatin.

**Supplementary Information:**

The online version contains supplementary material available at 10.1186/s13072-023-00491-w.

## Introduction

Eukaryotes effectively package their large genomes into the small volume of the nucleus by forming a condensed structure called chromatin. The basic repeating subunit of chromatin is the nucleosome core particle, composed of 146 bp of DNA wrapped around an octamer of the core histone proteins H3, H4, H2A and H2B [1, 2]. The histone N-terminal tails (NT) protrude from the nucleosome and interact with regulatory factors, including enzymes that add or remove various covalent post-translational modifications (PTMs) such as methylation, acetylation and phosphorylation [2–4]. Increasing evidence indicates that the establishment and maintenance of specific histone modification patterns, also known as the “histone code”, facilitates the regulation of DNA-templated processes at the corresponding locus, such as transcription, repair, replication and recombination [5, 6]. Perturbation of the dynamics of histone PTMs is directly related to certain developmental disorders and diseases, including cancer [7–9].

Proteolysis of the histone H3NT is another evolutionarily conserved histone modification in eukaryotes that results in the permanent removal of all existing histone PTMs on the H3NT. The cleaved H3 product (H3cl) is retained in ~ 5–10% of chromatin and can only be removed by histone replacement [9–13]. Although H3NT proteolysis was first described over 60 years ago, important biological insights into this fundamental epigenetic event have only occurred recently due to the discovery of several different histone proteases [14–22]. The lysosomal cathepsin L protease (CTSL) was the first H3NT protease discovered in differentiating mouse embryonic stem cells [14]. CSTL was reported to “clip” the first 21 amino acids from H3, generating a H3∆21 product within chromatin, in several different cell types including fibroblasts, melanocytes, developing brains and villi of small intestines [14, 18, 20, 23]. A recent study demonstrated that several neutrophil serine proteases coordinate H3NT proteolysis in monocytes resulting in multiple H3cl products [19]. In contrast, we previously discovered that the extracellular cellular matrix metalloproteinases 2 and 9 (MMP-2 and MMP-9) function as H3NT proteases during myogenesis and osteoclastogenesis, respectively, both generating a single H3∆18 product within chromatin [21, 22]. These collective findings indicate that different non-nuclear proteases are utilized as nuclear H3NT proteases in a cell type-specific manner. Although it is predicted that each protease is differentially targeted to distinct genomic regions to induce H3NT proteolysis, this has yet to be experimentally determined.

In this study we sought to discover the genomic targets of a known H3NT protease to gain new insights into the biological functions of H3NT proteolysis at these loci. To this end, we leveraged the recent finding that MMP-2 is the principal H3NT protease in the human U2OS osteosarcoma cell line with a native ChIP-Seq approach to map the genomic sites targeted for MMP-2 mediated H3NT proteolysis [24]. The results, which indicate that MMP-2 is selectively targeted to the TSSs of active genes, are consistent with a previous report demonstrating that MMP-2 mediated H3NT proteolysis facilitates gene activation during myogenesis [21]. One of the genes that required MMP-2 localization and H3NT proteolysis at TSS for proficient expression was the cathepsin B (CTSB) protease, which we discovered functions as an H3NT protease, generating two additional H3cl products at later time points during U2OS cell expansion in culture. The concerted actions of MMP-2 and CTSB mediated H3NT proteolysis resulted in a twofold reduction of histone H3NT PTMs at the TSSs of active genes. Our collective findings provide the first epigenomic map of an H3NT protease, further support a functional role for H3NT proteolysis in transcription, demonstrate that CTSB functions as an H3NT protease in cells, and that H3NT proteolysis profoundly alters the histone code at TSSs.

## Results

### U2OS cells display several H3NT proteolysis products during cell expansion

**Fig.1:**
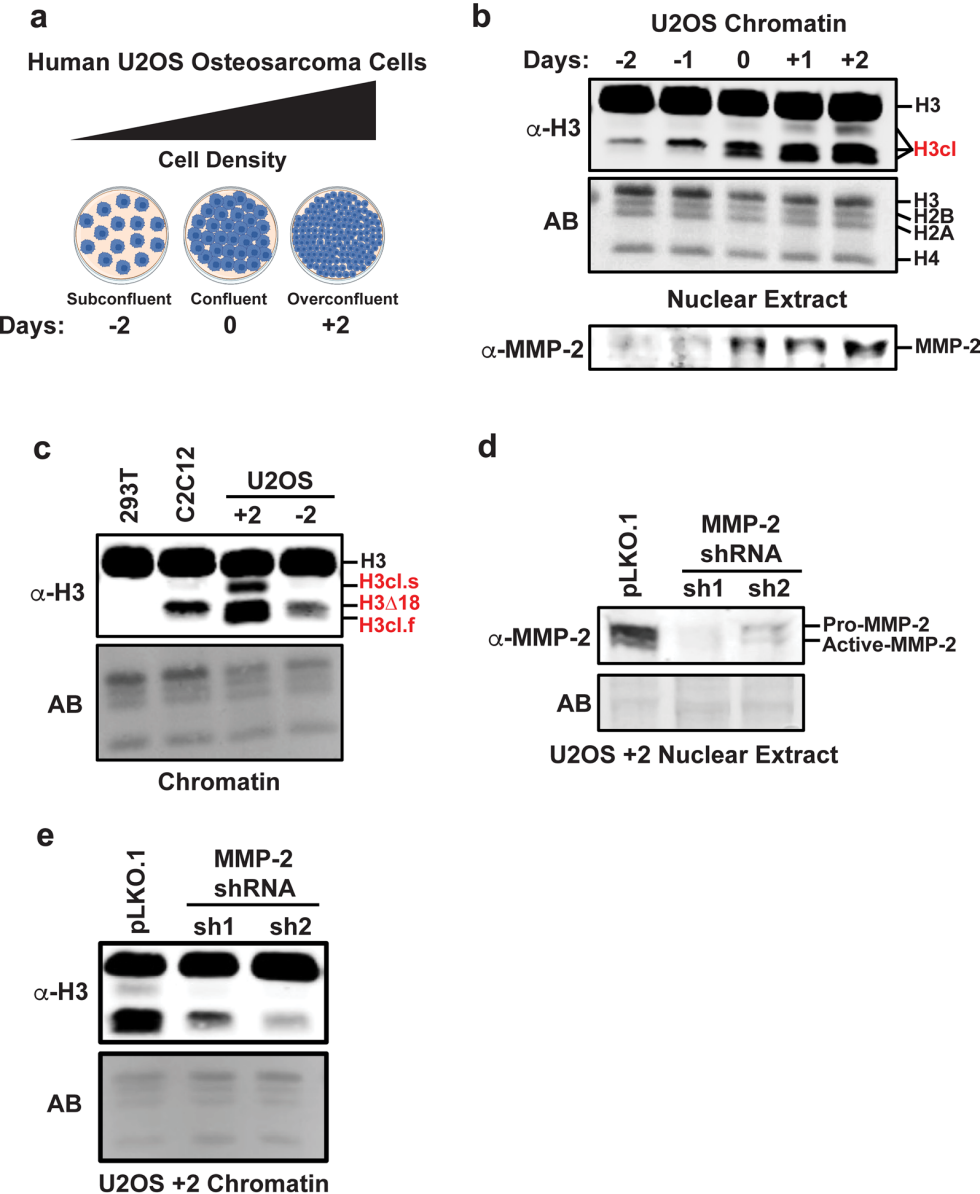
The MMP-2 protease directs H3NT proteolysis in U2OS cells. **a** Illustration of experimental time points examined in this study based on U2OS cell density, ranging from subconfluent (− 2 days) to confluent (0) to overconfluent (+ 2 days). **b** A histone H3 C-terminal antibody was used for Western analysis of chromatin purified from U2OS cells at the indicated time points. Proteolysis of the H3 N-terminus (H3NT) generates faster-migrating cleaved H3 (H3cl) products as indicated. Amido black stain (AB) of the membrane shows equivalent loading of chromatin between samples. Lower panel shows Western analysis of purified soluble nuclear extracts at these time points using an MMP-2 antibody. **c** Western analysis of purified chromatin from 293 T negative control cells, C2C12 differentiated myotube positive control, and U2OS + 2 and − 2 cells. The MMP-2 generated H3NT cleaved product (H3∆18) and two other H3 cleaved products, a slower migrating product (H3cl.s) and a faster migrating product (H3cl.f), are indicated. **d** Western analysis of nuclear extracts purified from stable U2OS + 2 cell lines expressing the pLKO.1 negative control or pLKO.1-MMP-2 shRNAs (sh1 and sh2) demonstrates depletion of the pro-form and catalytically active form of MMP-2. **e** Western analysis of chromatin from the same samples as in **d**

To examine the profile of H3NT proteolysis during the expansion of cultured U2OS cells, cells were seeded at low density and expanded before purifying chromatin extracts at various time points: 2 days prior to confluence (− 2), at confluence (0) and 2 days after confluence (+ 2) (Fig. 1a). Western analysis was performed using a C-terminal histone H3 antibody to detect wild type H3 and the faster migrating H3NT proteolysis products within chromatin [21]. Consistent with a recent report, a single H3NT cleaved product was detected in subconfluent cells that progressively accumulated during cell expansion (Fig. 1b) [24]. The size was consistent with the MMP-2 generated H3∆18 product reported in mouse C2C12 myotubes and previously in U2OS cells (Fig. 1c) [21, 24]. Two additional H3NT cleaved products, previously unreported, were also observed as cells reached confluence and continued to accumulate past confluence: a cleaved product slightly smaller than H3 (H3cl.s), indicating proteolysis within the first few amino acids of the H3NT, and a cleaved product slightly smaller than the MMP-2 H3∆18 product (H3cl.f), indicating proteolysis beyond H3Q19. These results confirm that H3∆18 is the predominant H3NT proteolysis product in U2OS cells and that the other H3NT cleaved products are generated at later stages of U2OS cell expansion.

### MMP-2 is the principal H3NT protease in U2OS cells

The increased mRNA expression and progressive accumulation of MMP-2 protein in the nuclear soluble fraction of expanding U2OS cells were concurrent with increased abundance of the H3∆18 product within chromatin, however, it remained unclear if the additional H3cl products in confluent U2OS cells were dependent on MMP-2 (Fig. 1b, Additional file 1: Fig. S1a, Additional file 2: Fig. S2a) [21]. To resolve this, U2OS cells were stably transduced with the pLKO.1 control or two different pLKO.1-MMP-2 shRNAs, sh1 and sh2, both resulting in significant reductions of MMP-2 mRNA and nuclear soluble protein in subconfluent (− 2) and overconfluent (+ 2) cells compared to control (Fig. 1d and Additional file 1: Fig. S1b–c). Although MMP-2 depletion did not significantly impair cell proliferation compared to control, Western analysis of chromatin confirmed that the depletion of MMP-2 was directly proportional with the reduction of all three H3cl products (Fig. 1e and Additional file 1: Fig. S1d–e). These results indicate that MMP-2 is the principal H3NT protease in U2OS cells and that MMP-2 is indirectly required for the generation of the H3cl.s and H3cl.f products observed in the overconfluent U2OS cells.

### Intracellular MMP-2 and H3NT proteolysis are required for proficient gene expression

**Fig. 2:**
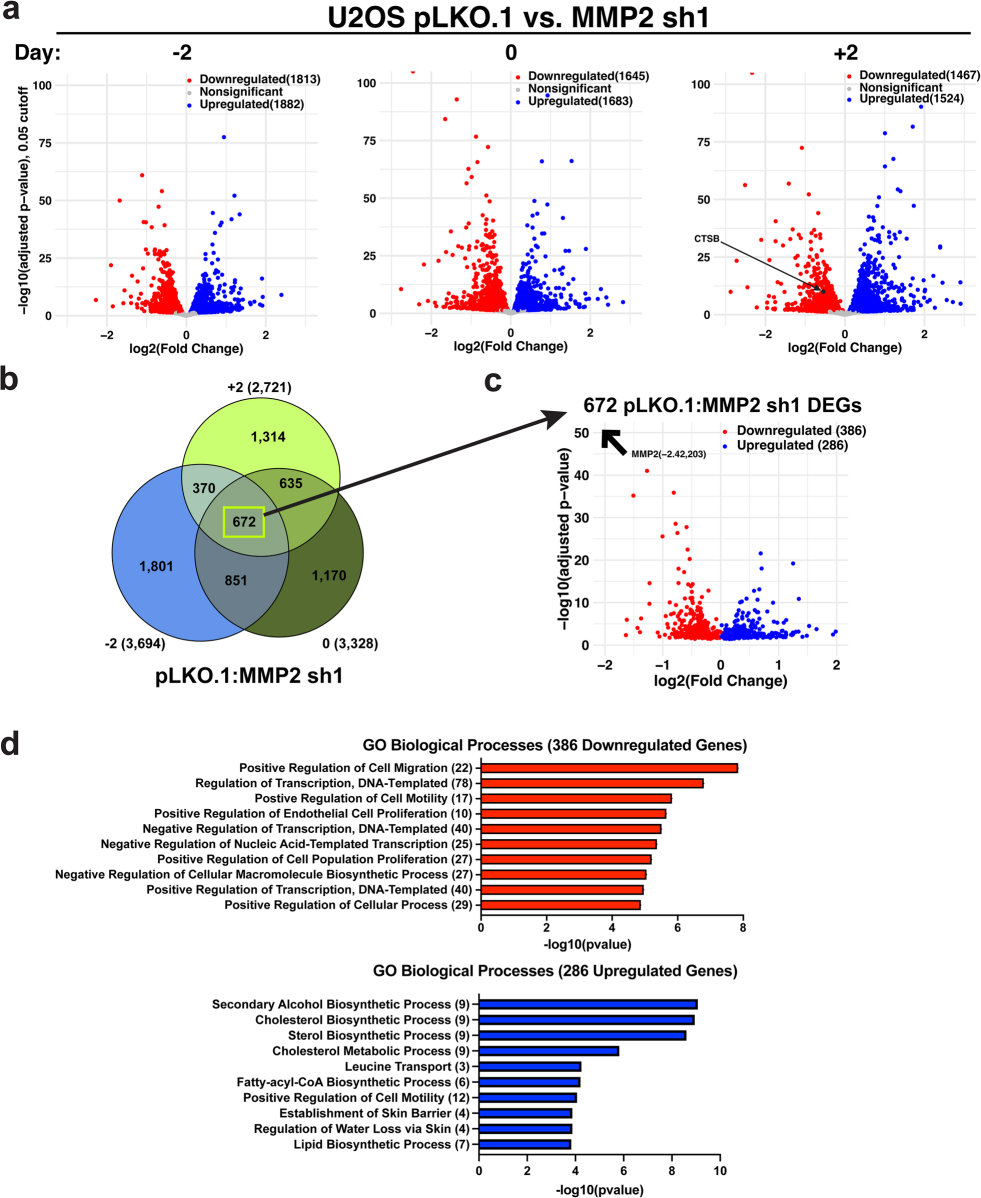
Intracellular depletion of MMP-2 and reduced H3NT proteolysis result in broad gene expression changes **a** Volcano plots generated from RNA-Seq data of U2OS pLKO.1 control versus MMP2sh1 subconfluent (− 2 days, left), confluent (0 days, middle) and over-confluent (+ 2 days, right) cells. The log adjusted fold change in expression (x-axis) was plotted relative to an adjusted p-value cutoff of 0.05 (y-axis). The number of significantly downregulated (red) and upregulated (blue) genes in the MMP2sh1 cells are indicated. **b** Venn diagram comparing all differentially expressed genes in the U2OS MMP2sh1 cells, relative to control, at the indicated time points. **c** Volcano plot of the 672 genes displaying significant expression differences throughout U2OS MMP2sh1 cell expansion relative to control. The average log adjusted fold change in expression (x-axis) was plotted relative to an average adjusted p-value (y-axis). The number of significantly downregulated (red) and upregulated (blue) genes are indicated. **d** Gene ontology (GO) of the downregulated (red) and upregulated (blue) genes from **c**. The top 10 terms (y-axis) and the number of genes in each term (parentheses) are plotted based on degree of significance (y-axis)

Previous reports support the role of the MMP-2 and MMP-9 H3NT proteases in the activation of lineage-specifying genes during myogenesis and osteoclastogenesis, respectively, suggesting that MMP-2 may also facilitate gene expression in U2OS cells [21, 22]. To identify the genes whose expression is dependent on MMP-2 and H3NT proteolysis, mRNA-Seq experiments were performed in the U2OS pLKO.1 control cells and the two U2OS pLKO.1-MMP-2 shRNA cells. Total RNA from three independent biological replicates of each of these stable cell lines were collected at the three different indicated times during cell expansion for mRNA purification and RNA-Seq (Fig. 2a and Additional file 2: Fig. S2d). A high degree of concordance between the replicates was observed, as well as a consistent reduction of MMP-2 mRNA in the MMP-2 shRNA replicates, relative to control (Additional file 2: Fig. S2b, c). Analysis of the data demonstrated that the expression of thousands of genes was significantly altered in MMP-2 depleted cells at each phase of U2OS cell expansion, relative to control. However, contrary to the hypothesis, there were no significant differences between the total number of genes that were aberrantly downregulated versus upregulated at each phase of cell expansion in the MMP-2 depleted cells (Fig. 2a and Additional file 2: Fig. S2d). Alternatively, additional meta-analysis between the time points was performed to identify the specific genes whose expression were constantly altered in cells with reduced MMP-2 and H3NT proteolysis (Fig. 2b). The results indicate only 672 differentially expressed genes were consistently altered across all phases of U2OS MMP2sh1 cell expansion, relative to control cells, with 386 genes (57%) displaying significantly decreased expression and 286 (43%) displaying increased expression (Fig. 2c). Gene ontology analysis revealed that genes constantly downregulated in MMP-2 depleted U2OS cells were most significantly related to proliferation, migration and transcriptional regulation (Fig. 2d). Conversely, upregulated genes were most significantly associated with metabolic and biosynthesis-related processes. Importantly, robust MMP-2 activity in the ECM was maintained in the U2OS MMP-2 shRNA cells due to abundant MMP-2 present in the cell growth media [21]. These collective results indicate that intracellular MMP-2 and H3NT proteolysis are required for the proficient expression of a subset of genes in U2OS cells, however, it remained unclear which of these genes were directly regulated by MMP-2 and H3NT proteolysis, and which were indirectly affected by MMP-2 depletion.

### MMP-2 is preferentially localized to 5’ transcription regulatory regions

**Fig. 3:**
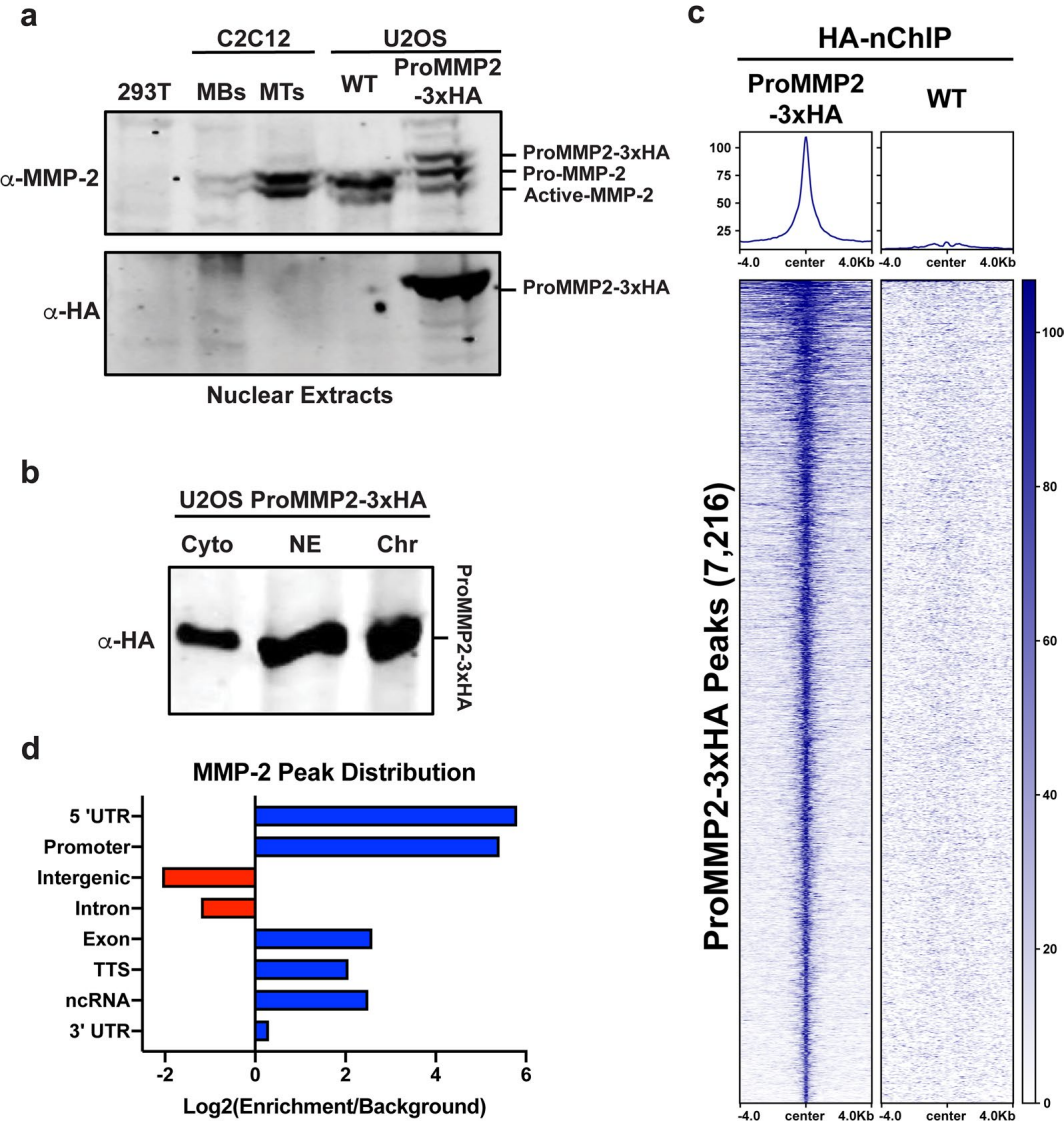
MMP-2 displays sharp chromatin binding at 5’ transcription regulatory regions **a** Western analysis of nuclear extracts purified from 293 T negative control cells, C2C12 myoblasts (MB) and differentiated myotubes (MT), and U2OS + 2 wild type (WT) and ProMMP2-3xHA cells using an MMP-2 (top) or HA antibody (bottom). ProMMP2-3xHA and the endogenous MMP-2 pro-form and catalytically active form are indicated. **b** U2OS + 2 ProMMP2-3xHA cells were fractioned into soluble cytoplasmic (Cyto) and nuclear (NE) extracts. The remaining insoluble chromatin (Chr) was washed repeatedly with 600 mM NaCl and solubilized via sonication prior to SDS-PAGE. Western analysis was performed with an HA antibody. **c** Heatmap and average plot profile of merged ProMMP2-3xHA and wild type (WT) negative control HA-nChIP-Seq replicates. RPKM normalized signal is plotted for each, centered over the 7216 ProMMP2-3xHA called peaks and extending ± 4 kb. **d** The genome wide distribution of the 7216 ProMMP2-3xHA peaks at defined genomic elements (y-axis) was determined using log2(peak enrichment/random background) values from the Homer annotatePeaks package (x-axis)

Since the genes directly regulated by MMP-2 and H3NT proteolysis would most likely be those occupied by MMP-2, ChIP-Seq experiments were performed to identify the specific genomic sites of MMP-2 localization in U2OS + 2 cells. ChIPs were initially performed using several commercially available MMP-2 antibodies but all were determined to be unsuitable for ChIP (data not shown). To overcome this technical barrier, a stable U2OS cell line was generated that constitutively expresses the full-length pro-form of MMP-2 with a C-terminal 3xHA epitope tag (ProMMP2-3xHA) (Additional file 3: Fig. S3a, b). Western analysis confirmed that the nuclear protein abundance of ProMMP2-3xHA was similar to that of wild type MMP-2 (Fig. 3a). Importantly, Western analysis of purified chromatin indicated that nuclear ProMMP2-3xHA remains tightly bound to chromatin, even after stringent high-salt washes (Fig. 3b). Due to the strong binding of ProMMP2-3xHA to chromatin, a native ChIP (nChIP) approach was utilized and demonstrated that a ChIP-grade HA antibody was successful in immunoprecipitating fragmented chromatin enriched in ProMMP2-3xHA (Additional file 3: Fig. S3c–e). Following this rigorous optimization, three independent biological HA-nChIP-Seq experiments were performed in the U2OS ProMMP2-3xHA cells and wild type control cells. A high degree of concordance was observed between the replicates, relative to control, with > 7700 peaks common between all replicates with a Pearson correlation of at least 0.85 (Additional file 3: Fig. S3f–h). The ProMMP2-3xHA peaks were strikingly narrow, spanning < 500 bp on average. The replicates were merged and re-analyzed resulting in the identification of 7216 ProMMP2-3xHA peaks across the genome (Fig. 3c). An unbiased annotation of the peak locations within the genome revealed a significant enrichment of ProMMP2-3xHA within promoter regions and 5’ UTRs, and a negative correlation with intergenic regions and introns (Fig. 3d). These findings indicate that ProMMP2-3xHA is preferentially localized to transcriptional regulatory regions in U2OS cells.

### MMP-2 is selectively targeted to the transcription start sites of protein coding genes

**Fig. 4:**
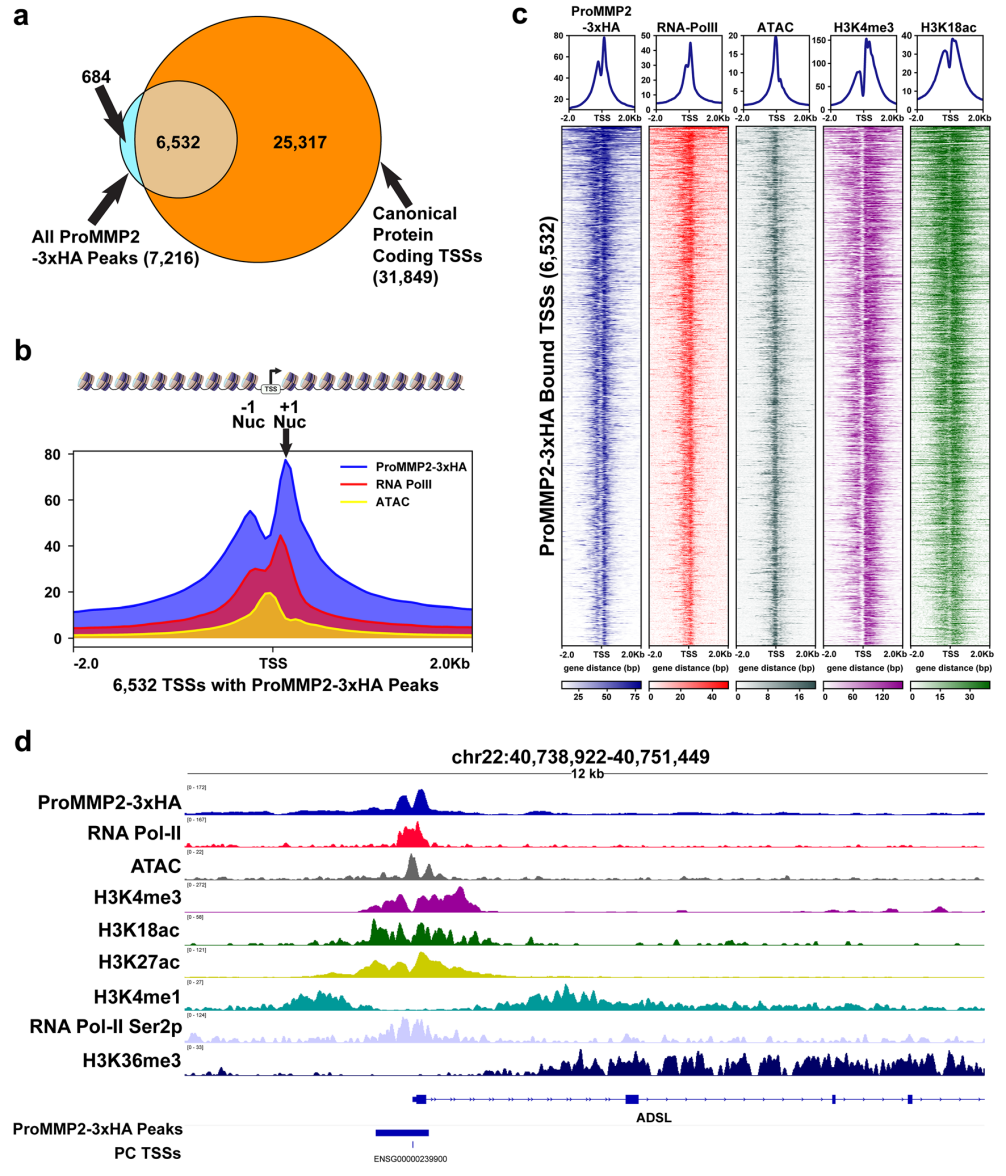
MMP-2 is specifically targeted to transcription start sites **a** Venn diagram showing overlap of ProMMP2-3xHA peaks within 1 kb of TSSs of all canonical protein coding genes. **b** Average peak profiles of the 6532 ProMMP2-3xHA peaks, RNA PolII and DNA accessible regions (ATAC) centered at TSSs. The hypothetical distribution of nucleosomes is depicted (top) over the ± 2 kb window (x-axis) versus average RPKM intensity (y-axis). **c** Heatmaps and average peak profiles of the RPKM intensity (y-axis) of ProMMP2-3xHA, RNA PolII, ATAC-Seq, H3K4me3 and H3K18ac centered at TSSs over a ± 2 kb window (x-axis). **d** Representative genome browser images of the ADSL gene. Distribution of ProMMP2-3xHA, RNA Pol II, ATAC-Seq and indicated H3 modification enrichments across the locus are displayed relative to the RPKM signal of each (y-axis). The ProMMP2-3xHA called peaks and TSSs are indicated (bottom)

Since the findings above suggested that MMP-2 is localized to the 5’ regulatory region of genes, the occupancy of ProMMP2-3xHA at all canonical protein coding genes was analyzed. Consistent with the results above, the analysis indicated that > 90% of ProMMP2-3xHA peaks were localized within 1 kb of the transcriptional start sites (TSSs) of protein coding genes (Fig. 4a). The average peak profile of ProMMP2-3xHA was plotted relative to the TSSs of these genes to examine the characteristics of localization at these regions. The results demonstrate that a clear primary site of enrichment is the first (+ 1) nucleosome after each TSS with a secondary site of enrichment at the -1 nucleosome (Fig. 4b). The narrow region of enrichment within these two nucleosomes is consistent with the average 488 bp peak size of ProMMP2-3xHA. Since this region spans the canonical nucleosome-free DNA accessible region of TSSs, it remained a possibility that ProMMP2-3xHA was non-specifically targeted to any DNA accessible region in the genome. To examine this, the > 43,000 DNA accessible regions in U2OS cells, as previously determined by ATAC-Seq, were analyzed for ProMMP2-3xHA occupancy [25]. While > 99% of ProMMP2-3xHA peaks are located within 1 kb of DNA accessible regions, as predicted, only 17% (7169) of all DNA accessible regions were occupied by ProMMP2-3xHA (Additional file 4: Fig. S4a). These collective data indicate that ProMMP2-3xHA is not arbitrarily targeted to DNA accessible regions, rather, is selectively targeted to the TSSs of a subset of protein coding genes.

### MMP-2 occupancy at TSSs is directly and positively correlated with gene expression

**Fig. 5:**
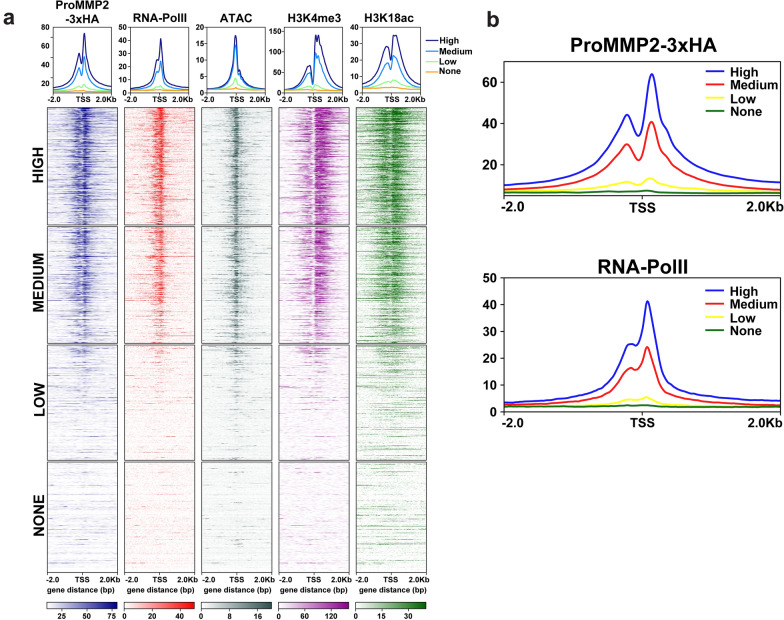
MMP-2 is selectively targeted to transcription start sites of active genes **a** Heatmaps and average peak profiles of the RPKM intensity (y-axis) of ProMMP2-3xHA, RNA PolII, DNA accessible regions (ATAC), H3K4me3 and H3K18ac centered at TSSs over a ± 2 kb window (x-axis). All protein coding genes were evenly separated into 4 groups based on the average transcripts per million, as indicated (high, medium, low or no expression). **b** Expanded average peak profiles of ProMMP2-3xHA (top) and RNA PolII (bottom), from **a**, plotting RPKM intensity (y-axis) centered at TSSs over a ± 2 kb window (x-axis)

The ProMMP2-3xHA peaks and peak profile were strikingly similar to those of RNA PolII at TSSs, as well as several histone H3 modifications associated with actively transcribed genes, suggesting a direct function of MMP-2 in facilitating transcription (Fig. 4c, d and Additional file 4: Fig. S4b, c) [25–28]. Therefore, it was predicted that the genes displaying reduced expression in the U2OS MMP-2 depleted cells would be selectively enriched in ProMMP2-3xHA at their TSSs compared to the genes that displayed increased expression (Fig. 2b, c). Although ProMMP2-3xHA was detected at the TSSs of most of the 672 differentially expressed genes, there was no clear correlation between ProMMP2-3xHA occupancy and gene expression (Additional file 5: Fig. S5a). Due to the small sample size of this analysis, an alternative unbiased approach was used to further examine the association between MMP-2 and gene expression in a broader context. Here, all protein coding genes were first evenly grouped based on their relative level of expression (none, low, medium and high), as determined by RNA-Seq in the U2OS + 2 cells, and then merged with the ProMMP2-3xHA peaks. The results indicate that ProMMP2-3xHA is almost exclusively targeted to the TSSs of high and medium expressed genes and, conversely, rarely detected at low or not expressed genes (Fig. 5a). Furthermore, increased abundance of ProMMP2-3xHA at TSSs was directly correlated with increased gene expression, analogous to that observed for RNA PolII (Fig. 5b). Similarly, co-occupancy of ProMMP2-3xHA and H3K4me3 at TSSs was typically associated with higher gene expression compared to H3K4me3 occupied genes with little to no ProMMP2-3xHA enrichment (Additional file 5: Fig. S5b–d). These collective results indicate that ProMMP2-3xHA occupancy at TSSs is directly and positively correlated to gene expression.

### Shorter genes display MMP-2 spreading into gene bodies

**Fig. 6:**
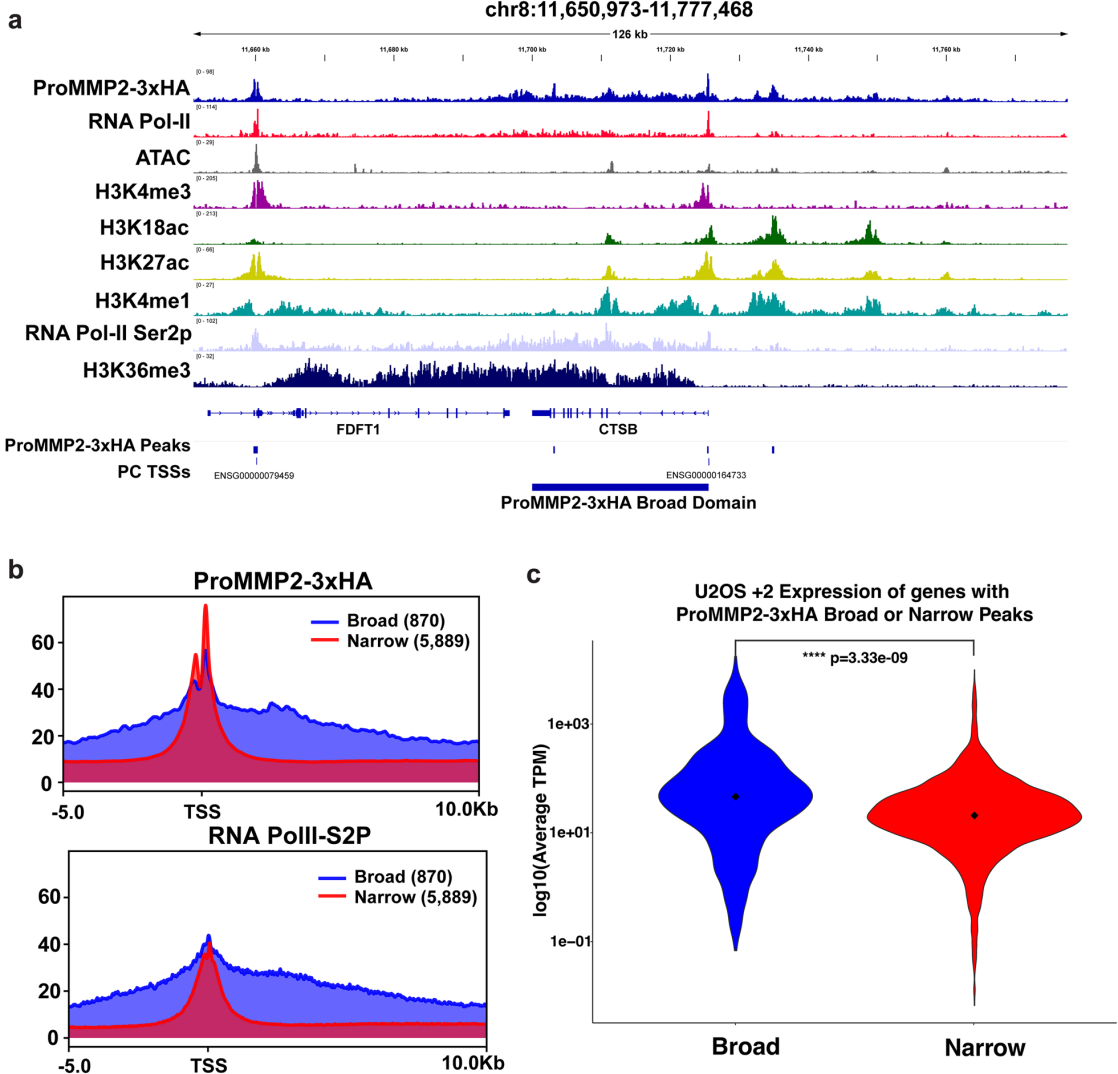
MMP-2 spreading into the bodies of active short genes **a** IGV genome browser snapshot of the FDFT1 and CTSB (Cathepsin B) genes that contain either a single sharp ProMMP2-3xHA peak at TSS or a broad region that extends from the TSS peak through the gene body, respectively (top tract). Other ChIP-Seq enrichment tracts for RNA PolII, DNA accessible regions (ATAC) and indicated histone H3 modifications are shown. The transcription start sites of protein coding genes (PC TSSs) and the ProMMP2-3xHA called peaks and identified broad regions are displayed (bottom). **b** The average RPKM ChIP-Seq signal intensities (y-axis) of the 5,889 ProMMP2-3xHA narrow peaks (red) and 870 broad peaks (blue), centered at transcription start site (TSS) and extending − 5/ + 10 kb (x-axis), were strikingly similar to that of the elongating form of RNA PolII (RNA PolII-S2P, bottom). **c** Violin plot comparing the log transformed average expression (transcripts per million (TPM), y-axis) of the 5889 ProMMP2-3xHA narrow peak genes (red) to the 870 broad peak genes (blue). A Wilcoxon rank sum test was used to determine statistical significance

Inspection of individual IGV tracts revealed that some ProMMP2-3xHA target genes displayed broader regions of ProMMP2-3xHA enrichment that spread beyond the major TSS peak (Fig. 6a). To investigate the general frequency of these broader regions of ProMMP2-3xHA enrichment, an unbiased analysis of the ChIP-Seq data was performed. While the results confirmed that the majority of the ProMMP2-3xHA positive regions identified were < 2 kb (13,423), there were also 3,194 ProMMP2-3xHA regions spanning 2–5 kb, 439 regions spanning 5–10 kb and 53 regions that were > 10 kb in length (Additional file 6: Fig. S6d). These findings indicate that a significant number of all ProMMP2-3xHA regions (> 21%) are longer than 2 kb, suggesting that a subset of ProMMP2-3xHA genes would also contain these broader regions. To examine this, the analysis was performed specifically at protein coding genes to identify those that contained ProMMP2-3xHA broad regions; defined as ProMMP2-3xHA positive regions covering at least 25% of the entire gene body. The analysis revealed that the vast majority of ProMMP2-3xHA target genes [5] had sharp narrow peaks focused at TSSs, as expected, but there were 870 genes that displayed significant broad regions of ProMMP2-3xHA enrichment beyond the TSSs (Fig. 6a, b and Additional file 6: Fig. S6a, b, e). Although the parameters of this biased analysis selects for shorter genes, the genes displaying broad ProMMP2-3xHA regions were typically expressed at higher levels compared to longer genes with narrow peaks, consistent with the positive correlation between MMP-2 abundance and gene expression described above (Fig. 6c and Additional file 6: Fig. S6c). The spreading of ProMMP2-3xHA within the bodies of these 870 genes was strikingly similar to the enrichment profile of elongating RNA PolII, further supporting a role for MMP-2 and H3NT proteolysis in transcription (Fig. 6b and Additional file 6: Fig. S6a)[27].

### Cathepsin B is the secondary H3NT protease in U2OS cells

**Fig. 7:**
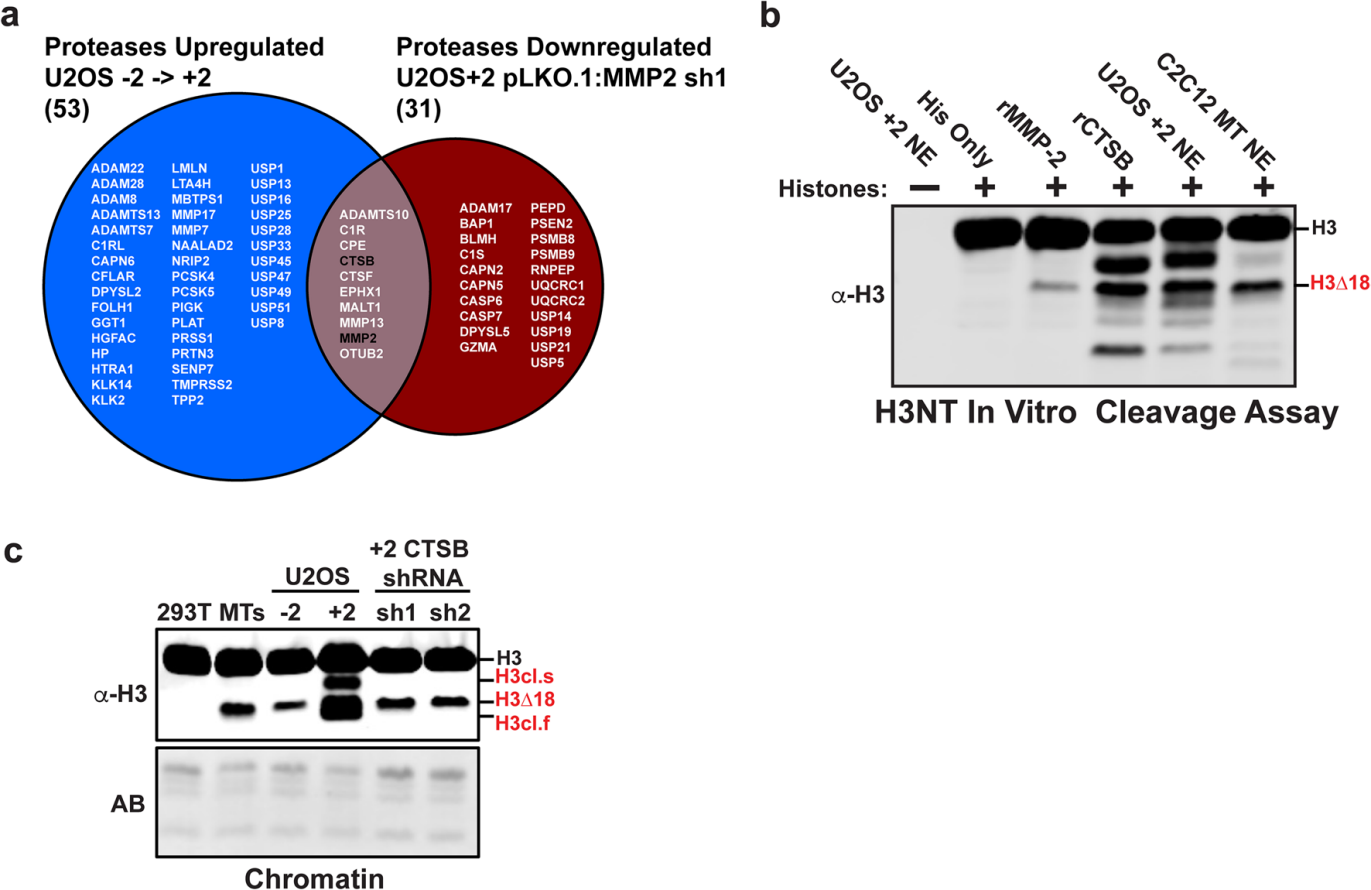
CTSB is the secondary H3NT protease in U2OS cells **a** Venn diagram comparing all known and predicted proteases that were upregulated during U2OS cell expansion (left) to those that were downregulated in U2OS + 2 MMP-2 depleted cells (right). MMP-2 and CTSB are highlighted in the overlapping list (middle). **b** In vitro H3 cleavage assay using core histone substrates incubated alone or with recombinant MMP-2 (rMMP-2) positive control, recombinant CTSB (rCTSB), or nuclear soluble extracts purified from U2OS + 2 cells or C2C12 differentiated myotubes (MT). Western analysis was performed to detect the H3NT cleaved products observed in U2OS + 2 chromatin (red). **c** Western analysis of purified chromatin from 293 T negative control cells, C2C12 differentiated myotubes (MT), U2OS pLKO.1 subconfluent (− 2) and over-confluent (+ 2) cells and U2OS + 2 cells expressing pLKO.1-CTSB shRNAs (sh1 and sh2). The MMP-2 generated H3∆18 product and two CTSB-generated H3NT cleaved products are indicated (red). Amido black stain (AB) of the membrane shows equivalent loading of chromatin between samples

One of the broad ProMMP2-3xHA genes identified above encodes the cathepsin B protease (CTSB) (Fig. 6a). Since previous reports demonstrated that other cathepsin proteases function as H3NT proteases, it was hypothesized that CTSB was the secondary H3NT protease responsible for generating the two additional H3cl products observed in U2OS + 2 cells (Fig. 1b) [14, 17]. To test this, an in silico analysis was first performed and confirmed that the top predicted sites of H3 proteolysis by CTSB were consistent with sizes of the H3cl products observed in the U2OS + 2 cells (Additional file 7: Fig. S7c, d) [29]. The hypothesis was further supported by analysis of the RNA-Seq data, demonstrating the CTSB was one of only 10 proteases that displayed increased expression during U2OS cell expansion and whose expression was significantly impaired in the U2OS + 2 MMP2sh1 cells (Figs. 2a, 7a and Additional file 7: Fig. S7a). To test this directly, in vitro H3 cleavage assays were first performed using core histone substrates and either recombinant CTSB or MMP-2 as the positive control, which generates the single H3∆18 product [21]. The results demonstrate that CTSB generates multiple H3NT cleaved products, and that the sizes of the CTSB-generated products were strikingly similar to those observed when U2OS + 2 nuclear extracts were used in the H3 cleavage assay (Fig. 7b). To validate these findings in cells, stable U2OS cell lines were generated expressing two different pLKO.1-CTSB shRNAs, sh1 and sh2, with both resulting in the significant reduction of CTSB mRNA in the cells (Additional file 7: Fig. S7b). Western analysis of purified chromatin demonstrated that CTSB depleted cells displayed a near ablation of the slowest (H3cl.s) and fastest (H3cl.f) migrating H3 cleaved products, however, the MMP-2 generated H3∆18 band was preserved (Fig. 7c). These collective findings indicate that CTSB is the secondary H3NT protease in U2OS cells that requires MMP-2 for activation at later stages of cell expansion.

### MMP-2 directs H3NT proteolysis at TSSs

**Fig. 8:**
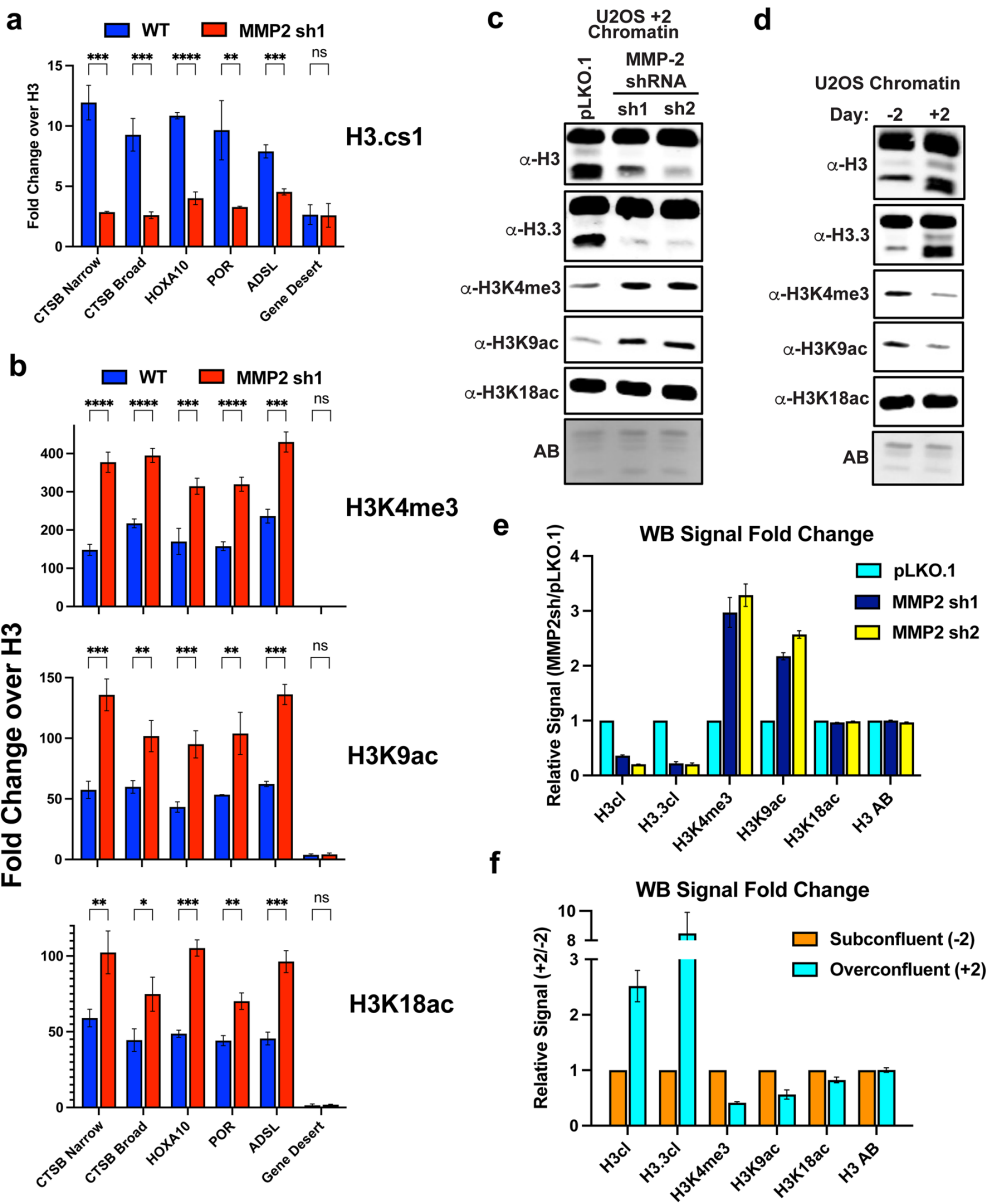
MMP-2 mediated H3NT proteolysis alters the histone code at TSSs **a** ChIP was performed in U2OS wild type (WT, blue) and MMP-2 depleted cells (MMP2sh1, red) using the H3.cs1 antibody, that detects cleaved H3NT, and a C-terminal H3 antibody to normalize for nucleosome occupancy. qPCR was performed at the indicated ProMMP2-3xHA positive gene loci and a negative control locus (gene desert, x-axis). Enrichment of cleaved H3NT at each locus was plotted as fold change relative to H3 control (y-axis), with the average and standard deviation between three independent biological replicates shown. Statistically significant differences between WT and MMP2sh1 at each locus were determined by a Student T-test (p-value: ns > 0.05, * < 0.05, ** < 0.005, *** < 0.0005, **** < 0.00005). **b** ChIP-qPCR analysis of histone H3 lysine 4 trimethyl (H3K4me3, top), lysine 9 acetyl (H3K9ac, middle) and lysine 18 acetyl (H3K18ac, bottom) as described in **a**. **c** Western analysis of chromatin purified from U2OS pLKO.1 control cells and the 2 different U2OS pLKO.1-MMP2 shRNA cell lines (sh1 and sh2) using the indicated antibodies. Amido black stain (AB) of the membrane shows equivalent loading of chromatin between samples. **d** Western analysis of chromatin purified from U2OS subconfluent (− 2) and overconfluent (+ 2) cells, as described in **c**. **e** Quantitative analysis of band intensities from **c** for each antibody (x-axis) was performed using ImageStudio (BioRad) and plotted as fold change relative to the pLKO.1 control, with the average and standard deviation of three independent biological replicates shown. **f** Quantitative analysis of band intensities from **d**, as described in **e**, plotted as fold change relative to U2OS subconfluent cells (− 2)

While it was predicted that the genomic loci selectively targeted for ProMMP2-3xHA localization would also likely be targeted for H3NT proteolysis, this had yet to be experimentally validated. To examine this directly, an antibody capable of binding cleaved H3 (H3.cs1) in ChIP-qPCR experiments was used with purified chromatin from U2OS + 2 wild type cells [14, 19]. Three ProMMP2-3xHA genes displaying narrow peaks at TSSs, the CTSB gene at both the narrow TSS peak and broad region within the gene body, and a gene desert negative control region were queried. As predicted, the results demonstrate a significant enrichment of cleaved H3, relative to overall histone H3 occupancy, at the ProMMP2-3xHA loci compared to the negative control region (Fig. 8a and Additional file 8: Fig. S8b). Importantly, depletion of MMP-2 and H3NT proteolysis in the U2OS + 2 MMP2sh1 cells resulted in a two-fivefold reduction of cleaved H3 at these loci compared to wild type control. These data confirm that MMP-2 induces H3NT proteolysis specifically at the target loci and, furthermore, demonstrates that ChIP-Seq of an H3NT protease can proxy for identification of the genomic sites targeted for H3NT proteolysis.

### H3NT proteolysis causes local and global reductions in H3NT PTMs at TSSs

The findings above predict that MMP-2 mediated H3NT proteolysis at TSSs would cause a proportional reduction of canonical promoter-specific H3 PTMs, including H3K4me3, H3K9ac and H3K18ac [30]. To test this, ChIP-qPCR was repeated at the ProMMP2-3xHA target TSSs above in U2OS wild type and MMP2sh1 cells. As predicted, the fold-reduction of cleaved H3 at each locus in the U2OS MMP2sh1 cells, relative to wild type, was strikingly proportional to the observed fold-increase of H3K4me3, H3K9ac and H3K18ac at the respective locus (Fig. 8a, b). Since H3K4me and H3K9ac are selectively localized at TSSs, these findings predicted that global increases in these modifications would also be observed following depletion of H3NT proteolysis. To test this, Western analysis of chromatin from U2OS + 2 MMP-2 shRNA and wild type control cells was performed (Fig. 8c). Consistent with the ChIP-qPCR results, the U2OS MMP-2 shRNA cells displayed a > twofold increase in global H3K4me3 and H3K9ac abundance compared to control (Fig. 8e). Significant changes in H3K18ac were not observed due to the localization of this modification at genomic regions besides TSSs (Additional file 8: Fig. S8a). These findings predicted that the induction of H3NT proteolysis observed in expanding U2OS cells would similarly result in global reductions of H3K4me and H3K9ac. To test this, Western analysis of chromatin from U2OS subconfluent (-2) and over-confluent (+ 2) cells that display low and high amounts of cleaved H3, respectively, was performed (Fig. 8d). The results confirmed that increased H3NT proteolysis in the U2OS + 2 cells resulted in a ~ twofold global reduction of H3K4me3 and H3K9ac compared to the U2OS -2 cells (Fig. 8f). These collective results indicate that the programmed induction of MMP-2 dependent H3NT proteolysis in expanding U2OS cells results in reduced H3K4me3 and H3K9ac at the TSSs of active genes.

## Discussion

Although “clipping” of the histone H3NT is a conserved epigenetic feature in most eukaryotes, the specific genomic sites targeted for H3NT proteolysis and the functional consequence of this modification remain largely unknown. Progress in this field has been hindered due to the lack of rigorous approaches to identify the genomic locations of the various cleaved H3 products. To overcome this technical barrier, we used an alternative strategy in this study to map the nuclear MMP-2 H3NT protease, as a proxy for the genomic locations of H3∆18, to gain new insights into the functions of H3NT proteolysis. To this end, the native ChIP-Seq technique was used to map MMP-2, in part, because nuclear MMP-2 was found to bind chromatin with high affinity. This has similarly been reported for other H3NT proteases, including CSTL, MMP-9 and the neutrophil serine proteases, supporting high affinity chromatin binding as a conserved property of H3NT proteases [19, 20, 22, 31]. This is an unexpected trait since the canonical functions and substrates of all these proteases are extra-nuclear and, furthermore, that the proteases lack any canonical DNA binding domains or nuclear protein–protein interaction domains. The mechanisms that facilitate nuclear localization of the H3NT proteases and their selective targeting to specific genomic sites remain unknown.

The native ChIP-Seq results indicate that MMP-2 is selectively targeted to TSSs of protein coding genes with the primary site of enrichment at the + 1 nucleosome. Importantly, ChIP-qPCR confirmed that the identified MMP-2 occupied sites also displayed specific enrichment for cleaved H3, demonstrating that ChIP-Seq of an H3NT protease can proxy to identify the genomic sites of cleaved H3. Our findings that MMP-2 and H3NT proteolysis are selectively targeted to the TSSs of active protein coding genes are strikingly similar to previous reports. In osteoclasts, the TSSs of osteoclastogenic genes displayed H3NT proteolysis and, in monocytes, the H3∆21 product was enriched at the TSSs of active genes [19, 22]. While these findings support TSSs as the primary target site of H3NT proteases, it remains unclear whether distinct H3NT proteases and the H3cl products that they generate are targeted to distinct TSSs in different cell types.

MMP-2 was localized almost exclusively to the TSSs of highly expressed genes, supporting a direct role for MMP-2 mediated H3NT proteolysis in regulating transcription. This is consistent with previous reports demonstrating that H3NT proteolysis facilitated the activation of lineage-specifying genes during myogenesis and osteoclastogenesis [21, 22]. Although these reports predicted that the MMP-2 target genes in U2OS cells would be downregulated following depletion of MMP-2 and H3NT proteolysis, there were only slightly more MMP-2 target genes that were downregulated (57%) than upregulated (43%). Interestingly, these findings were consistent with other reports where the proportions of differentially expressed genes in melanoma cells and lymphoma-derived monocytes following depletion of H3NT proteolysis were strikingly similar, with 57% of genes downregulated and 43% upregulated [19, 32]. These collective findings suggest that the establishment of H3NT proteolysis at lineage-specifying genes is necessary for their activation and proficient terminal differentiation, whereas H3NT proteolysis plays a minor role in the maintenance of target gene expression in proliferating cancer cells.

CTSB was one of the genes displaying extensive H3NT proteolysis that required MMP-2 for activation. Although CTSB was previously reported to cleave histone H3 in vitro, consistent with our results, we demonstrate that CTSB functions as an H3NT protease in confluent U2OS cells [9]. The cell density-dependent activation of CTSB and the cleaved H3 products generated are similar to those observed by CSTL during cellular senescence [23]. These findings suggest that cathepsin-mediated H3NT proteolysis may play a functional role in facilitating cell cycle exit and/or maintenance of quiescence. It currently remains unknown whether CTSB is primarily targeted to distinct genomic sites, independent of MMP-2, or if CTSB is primarily localized to MMP-2 occupied sites, thereby, reinforcing H3NT proteolysis at the TSSs of active genes.

Our results demonstrate that H3NT proteolysis results in the global and local reductions of promoter-specific histone H3 PTMs, including H3K4me3 and H3K9ac. Similar outcomes were reported during the programmed induction of H3NT proteolysis in differentiating cells, suggesting that any observed reductions in histone H3 PTMs may be a general indicator of H3NT proteolysis in different cell types [18, 33]. Although H3NT proteolysis resulted in a > twofold reduction in H3K4me3, H3K9ac and H3K18ac, the significant enrichment of these histone H3 PTMs at TSSs persisted. One possible explanation for these findings is that only one of the H3NTs per nucleosome is targeted for proteolysis, thereby generating an asymmetric cleaved nucleosome at TSS [34]. Alternatively, the H3NT protease may indiscriminately cleave both H3NTs, generating a symmetric cleaved nucleosome, but the rapid exchange and modification of wild type histone H3 observed at TSSs masks the detection of this event by ChIP [35]. Further investigation is required to resolve these possibilities. Regardless, the functional significance of H3NT proteolysis and resulting reduction in the activation-associated H3 PTMs at TSSs of active genes remain unclear. A recent report demonstrated that H3K4me3 regulates RNA PolII promoter-proximal pause-release, suggesting that H3NT proteolysis may indirectly regulate RNA PolII function by modulating the levels of H3K4me3 at TSSs [36]. In addition, H3NT proteolysis may function directly to facilitate the localization and retention of RNA PolII by clearing bulky H3 PTM-specific binding proteins and complexes from TSSs [37]. Likewise, several biophysical studies demonstrated that lack of the H3NT destabilizes intra- and inter-nucleosome interactions that impair formation of a higher order solenoid chromatin structure, suggesting that H3NT proteases may directly function as ATP-independent chromatin remodelers to increase the accessibility of transcription factors and RNA PolII at TSSs [38–46]. Although these mechanistic possibilities are not mutually exclusive, further investigation is required to determine how H3NT proteolysis specifically functions in transcriptional regulation.

## Conclusions

This study demonstrates that H3NT proteolysis occurs in a cell density dependent manner in U2OS osteosarcoma cells, by the primary protease MMP-2 and the secondary and novel protease CTSB. The study provides the first genome wide binding of an H3NT protease, showing it occurs at all active TSSs narrowly at the + 1 nucleosome. MMP-2 mediated H3NT proteolysis at the CTSB gene locus mediates its upregulation, and as a result additional H3cl products are produced by CTSB. Together, this leads to a local and global reduction of active histone H3 PTMs at select TSSs, which results in modest changes in gene expression at a small subset of genes across all three stages of U2OS growth.

## Methods

### Cell culture

U-2 OS cells (ATCC) were grown in DMEM (Corning 10–013-CV) supplemented with 10% FBS (ThermoFisher, 26,140,079), 1X GlutaMAX (ThermoFisher, 35,050), 1X antibiotic–antimycotic (ThermoFisher 15,240) and 1X Non-Essential Amino Acids (ThermoFisher, 11,140,050). Cells were plated at 30% confluence (Day -2) and grown to reach confluence (2 days later, day 0) or allowed to continue to grow to become over-confluent (2 additional days, day + 2).

### Cytoplasmic extraction, nuclear extraction and chromatin purification

Isolation of cytoplasmic extracts, nuclear extracts, and purified chromatin was performed as previously described [47]. Cells were harvested, washed in PBS, and resuspended in low salt buffer (LSB) (20 mM HEPES pH 7.9, 25% glycerol, 1.5 mM MgCl_2_, 2 mM EDTA, 1 mM DTT, Halt Protease and Phosphatase Inhibitor Cocktail). Cells were incubated on ice for 15 min to allow the cells to swell. Cells were lysed by adding the non-ionic detergent NP-40 (0.75% final) and gently passed through a 21-gauge needle ten times. Nuclei were collected by spinning at 1100 × g at 4 °C for 5 min. The supernatant was collected as the cytoplasmic extract. Nuclei were washed twice with LSB and then resuspended in 500 µLs of LSB. The pelleted nuclear volume (PNV) was determined by measuring the total volume—500 µL LSB. Nuclei were repelleted and resuspended in ½ PNV of the LSB. ½ PNV of high salt buffer (HSB) (20 mM HEPES pH 7.9, 25% glycerol, 1.5 mM MgCl_2_, 1.6 M NaCl, 1 mM DTT, Halt Protease and Phosphatase Inhibitor Cocktail) was added dropwise while vortexing at a low speed to achieve a final NaCl concentration of 400 mM. Samples were incubated at 4 °C while shaking for 1 h before centrifuging them at 21,000 × g for 10 min at 4 °C. The supernatant was collected as the soluble nuclear extract. The pellet (chromatin fraction) was washed twice for 10 min with shaking the 400 mM NaCl HSB, pelleted, and resuspended in 1X SDS-PAGE load dye (final: 50 mM Tris–HCl pH 6.8, 3% SDS, 10% glycerol, 5% β-mercaptoethanol, 0.002% bromophenol blue). Samples were boiled at 95 °C for 5 min and cooled on ice three times before shearing the chromatin by sonication (15 s using the Misonix Sonicator3000 (Power setting 3.0), repeated until pellet dissipated completely). The cytoplasmic and nuclear soluble extracts were quantitated using the BCA Protein Assay Kit (ThermoFisher 23,225) prior to loading for Western analysis.

### Western blot analysis

Chromatin samples were fractionated on a 15% SDS-PAGE gel (cast in house), transferred to a 0.2 µm nitrocellulose membrane (GE Healthcare, GE10600001) using Towbin buffer (25 mM Tris pH 8.8, 192 mM Glycine, 20% methanol) on a Hoefer TE 77 semi-dry transfer unit for 1.5 h at 55 mA/membrane. 20–30 µgs of cytoplasmic and nuclear soluble extracts were fractionated on a 10% SDS-PAGE gel and transferred to a 0.45 µm nitrocellulose membrane using the same buffer and transfer unit. Each membrane was incubated in a blocking solution (4% non-fat milk in TBS) for 1 h prior to replacement with primary antibody diluted in 1.5% milk in TBS. Antibodies and the concentrations used are supplied in Additional file 9: Table S6. Membranes were incubated while rocking in the primary antibody overnight at 4 °C, washed in TBS-T (TBS, 0.1% Tween-20) three times for 10 min and incubated in TBS-T with 1.5% milk with a secondary antibody for 45 min while covered at room temperature. The membrane was then washed three times for 10 min in TBS-T, rinsed in diH2O and imaged at 700 nm using the Odyssey^®^ CLx imaging system (LI-COR). Equivalent loading was confirmed by staining the membrane following imaging with Amido Black stain (10% acetic acid, 0.1% Amido Black 10B (w/v)).

### Viral plasmid transfections

Half a million 293 T cells (ATCC) were plated in a 6-well plate 24 h prior to transfection. A transfer plasmid, psPAX2, and pMD2.G were transfected at a 4:3:1 ratio into the 293 T cells using Lipofectamine 3000 according to the manufacturer’s protocol (ThermoFisher, L3,000,001). Fresh media was replaced the following day, and after 72 h media containing virus was collected.

### Lentivirus transductions

200 k U2OS cells were plated in a 6-well plate 24 h prior to transduction. Virus containing media was filtered using a 0.45 µm cellulose acetate membrane (VWR 28145–481) and 10 µg/mL of Polybrene (Millipore, TR-1003-G) was added. Viral media was added to the cells and a spinfection performed at 1100 × g for 1 h at 33 °C. Fresh media was added 24 h later. Puromycin was added at a concentration of 2 µg/mL for 5 days with replacement every 2 days. shRNA plasmids were obtained as glycerol stocks from Sigma and grown for midipreps using the PureLink HiPure Plasmid Midiprep Kit (ThermoFisher, K210004). The following shRNA’s were used in this study: MMP2 (**TRCN0000051523** and **TRCN0000051526**), CTSB (**TRCN0000003655** and **TRCN0000003658**), and pLKO.1 control (**SHC001**).

### RNA isolation

Total RNA was extracted using TRIzol reagent according to the manufacturer’s protocol (ThermoFisher, 15,596) Briefly, cells were washed with PBS briefly before adding 1 mL of TRIzol directly to cells. Cells were scraped and the mixture transferred to a microfuge tube. Chloroform was added at a ratio of 1:5 (v/v) and the samples were vortexed vigorously and centrifuged at maximum speed at room temperature for 15 min. The RNA-containing aqueous phase was isolated and combined 1:1 (v/v) with Isopropanol at room temperature. RNA was pelleted at max speed at 4 °C and washed twice with 70% ethanol. The dried pellet was then resuspended in nuclease free diH_2_O and RNA concentration determined by spectrophotometry.

### RT-qPCR

One µg of total RNA was reverse transcribed using the SuperScript IV VILO Master Mix according to the manufacturer’s protocol (ThermoFisher, 11,756). 10 ng’s of the resulting cDNA was used for qPCR using the PerfeCTa^®^ SYBR^®^ Green FastMix^®^ (QuantaBio, 95,072)) according to the manufacturer’s protocol in an iQ5 iCycler (BioRad). 5 µM each of the forward and reverse primer were used (see Additional file 9: Table S7). Quality and specificity of primer sets were validated by melt curve analysis. Expression of each gene was calculated as 2^(− ∆∆Ct) relative to the GAPDH housekeeping gene. Three independent biological replicates were performed to determine the average expression change and standard deviation.

### In vitro H3 cleavage assay

Nuclear soluble extracts (3 μgs) or recombinant protein (100 ngs) were incubated with acid extracted core histones purified from C2C12 myoblasts (1 μg) in Cleavage Buffer (10 mM Tris–HCl pH 7.5, 10 mM KCl, 1.5 mM MgCl_2_, 1 mM CaCl_2_) at 37 °C for 1 h [23]. Reactions were quenched with 6X SDS-load dye and boiled for 5 min. SDS-PAGE and Western analysis was performed as described above.

### RNA-seq

RNA-seq was performed by Novogene Corporation. Total RNA quality was assessed using an Agilent TapeStation RNA ScreenTape (Agilent, 5067–5576) and only samples with a RIN score above 7 were used for sequencing. mRNA was selected using the NEBNext Poly(A) mRNA Magnetic Isolation Module (NEB, E7490), and cDNA synthesis and libraries made using the NEBNext Ultra II RNA Library Prep Kit for Illumina (NEB, E7770) according to the manufacturers protocol. Libraries were sequenced on a NovaSeq 6000 System using an S1 Flow Cell (Novagene Corporation) in PE 2 X 150 bp mode. Over 20 million reads were obtained for each sample.

### MNase digestion of chromatin

Native ChIP-seq was adapted from [48]. 2.85 Units of micrococcal nuclease (MNase) per million nuclei from Worthington (LS004797) was used for all experiments. Briefly, cells were washed with ice-cold PBS before scraping and collecting them. Cells were kept on ice and all spins done at 4 °C unless otherwise noted. All buffers had Halt Protease and Phosphatase Inhibitor Cocktail and 10 mM Sodium Butyrate added. Cells were resuspended in native ChIP Lysis Buffer (NLB) (10 mM Tris–HCl pH 8.0, 10 mM NaCl, 3 mM MgCl_2_, 0.1% NP-40) and lysed by gently passing them through a 21-gauge needle ten times. Nuclei were collected by spinning at 1100 × g for 5 min, and supernatant discarded. Nuclei were resuspended in NLB, quality assessed and counted using 0.2% Trypan Blue (ThermoFisher, 15,250,061). 40 million nuclei per cell line were resuspended in 500 µLs of MNase Digestion Buffer (MDB) (10 mM Tris–HCl pH 8.0, 10 mM NaCl, 3 mM MgCl_2_, 0.1% NP-40, 250 mM Sucrose, 3 mM CaCl_2_). Nuclei were incubated at 37 °C for 5 min, and then MNase added (2.85 U/1 million nuclei). Nuclei were incubated at 37 °C and lightly vortexed every minute for 4 min. MNase was quenched using EDTA (4 mM final concentration) and nuclei incubated on ice for 10 min. Nuclei were pelleted at 1100 × g for 5 min and the supernatant saved as the soluble 1 (S1) fraction. The remaining nuclei were resuspended in 300 µLs of native ChIP release Buffer (NRB) (10 mM Tris–HCl pH 7.5, 10 mM NaCl, 0.2 mM EDTA) and incubated on a nutator for 1 h at 4 °C. The nuclei were centrifuged at max speed for 10 min at 4 °C and the supernatant collected as the soluble 2 (S2) fraction. The remaining pellet (P) was washed once with NRB and then frozen for further analysis.

### Native ChIP

The S1 and S2 fractions were combined and diluted 10X in native ChIP Incubation Buffer (NIB) (10 mM Tris–HCl pH 7.5, 70 mM NaCl, 2 mM MgCl_2_, 2 mM EDTA, 0.1% Triton X-100). The lysate was pre-cleared with 60 µL of Pierce ChIP-Grade Protein A/G Magnetic Beads (ThermoFisher, 26,162) with rotating at 4 °C for one hour. Beads were discarded and lysate divided up. Histone ChIPs used 50 µgs of chromatin (determined from collecting S1/S2 pooled input and isolating DNA for quantification) and HA ChIP used 200 µgs of chromatin. Antibodies were added for each IP (See Additional file 9: Table S6 for amounts used) and rotated end-over-end overnight at 4 °C. 25 µLs of magnetic beads per IP were washed 3X in NIB and blocked in 1% BSA overnight. The following morning beads were washed 3X with NIB and resuspended in their original volume. 25 µLs of blocked beads were added to each IP for three hours. Each lysate was washed 4X in native ChIP Wash Buffer (NWB) (20 mM Tris–HCl pH 7.5, 135 mM NaCl, 2 mM EDTA, 0.1% Triton X-100) with 5 min of rotation at 4 °C between each wash. Lysates were then washed 2X in native ChIP LiCl Wash Buffer (LWB) (10 mM Tris–HCl pH 7.5, 250 mM LiCl, 1 mM EDTA, 0.5% NP-40, 0.5% Na-DOC) with 5 min of rotation at 4 °C. Lysates were then washed once in TE Wash Buffer (TWB) (50 mM Tris–HCl pH 8.0, 10 mM EDTA), followed by a short spin to get rid of all residual buffer. Beads were resuspended in 200 µL of Elution Buffer (EB) (1% SDS, 0.1 M NaHCO3) and shaken at 800 RPM at 37 °C for 30 min. A final elution of 10 min at 65 °C was then performed. Supernatant was collected and RNase A (2 µgs, ThermoFisher, R1253) and Proteinase K (5 µgs, VWR, E195) were added and incubated at 37 °C for 30 min and then 55 °C for 3 h. 200 µL of Phenyl:Chloroform:Isoamyl Alcohol (25:24:1, Sigma, P2069) was added and samples vortexed for 15 s, and then centrifuged at max speed at room temp for 15 min. The aqueous phase was collected and 2.5X volumes 100% Ethanol, 10% (v/v) 3 M Sodium Acetate, and 3 µgs of Glycogen (VWR, N632) were added, vortexed, and incubated at − 80 °C for 1 h. Samples were centrifuged for 30 min at max speed at 4 °C, washed with 70% ethanol, and re-pelleted. Dried pellets were resuspended in 50 µL of nuclease free water. DNA was stored at − 20 °C until used for library prep or qPCR.

### ChIP library prep

Isolated ChIP DNA was used to make libraries using the NEBNext Ultra II DNA Library Prep Kit for Illumina (NEB, E7645S). Libraries were constructed according to the manufacturers protocol. Briefly, DNA was subjected to end-repair and dA-tailing. NEBNext adaptors (0.6 µM) were ligated onto DNA fragments, and then U excision performed by the USER enzyme. Size selection was performed using AMPure XP (Beckman Coulter, A63880) to get rid of additional adaptors. Amplification was performed using the NEBNext Ultra II Q5 Master Mix, the universal i5 primer and one index i7 primer. The appropriate cycles were determined by including 1X EvaGreen Dye (Biotium, 31,000) and stopping the reaction once the library reached 3000 RFU. Following PCR, a double-sided side selection was performed using AMPure XP beads (0.9–0.55) to get rid of residual primers and any large DNA fragments. Library quality and concentration was assessed using an Agilent Bioanalyzer and the HS DNA Kit (Agilent, 5067–4626). Libraries were sequenced on a NovaSeq 6000 System using an S1 Flow Cell (Novagene Corporation) in PE 2 X 150 bp mode. Over 30 million reads were obtained for each library.

### ChIP qPCR

1 µL of isolated ChIP DNA was used with 500 nM primers (both forward and reverse) and with PerfeCTa^®^ SYBR^®^ Green FastMix^®^ (QuantaBio, 95,072) according to the manufacturer’s protocol in an iQ5 iCycler (BioRad). All primer sequences can be found in Additional file 9: Table S8. Fold change over H3 was calculated as 2^-(IPct-H3IPct). 4 technical replicates were performed for each biological replicate. At least two biological replicates were done for each ChIP-qPCR. Statistical significance was calculated using a student T-test in Prism Graph Pad.

### Sequencing data analysis

All ChIP-seq and ATAC-seq data was processed through a modified version of the ENCODE pipeline (https://www.encodeproject.org/data-standards/chip-seq/) [49]. MACS2, bedtools and deepTools were used for peak calling, comparing datasets and visualization respectively [50–52]. Detailed methods of all analysis is provided in the supplemental methods. RNA-seq data was analyzed using DEseq2 and RSEM according to their documentation [53, 54]. ggplot2 was used for making volcano, violin, and boxplots [55]. Tidyverse and dyplr were utilized for merging ChIP-seq and RNA-seq datasets [56, 57]. Detailed methods of all analysis are provided in the supplemental methods Additional file 10.

## Supplementary Information

**Additional file 1: Figure S1.** **a** Western analysis of cytoplasmic and nuclear extracts purified from 293T negative control cells, C2C12 myoblast and myotube positive control, and U2OS +2 cells. An MMP-2 antibody detects the pro-form and catalytically active forms of MMP-2, as indicated. **b** RT-qPCR analysis of total RNA purified from cells stably transduced with either a pLKO.1 control or two different pLKO.1-MMP-2 shRNAs (sh1 and sh2). MMP-2 expression was normalized to GAPDH control and plotted relative to the pLKO.1 control (y-axis). Three independent biological replicates were performed to generate standard deviation (error bars). **c** Western analysis of nuclear extracts purified from subconfluent (-2) U2OS pLKO.1 negative control or two different pLKO.1-MMP-2 shRNAs (sh1 and sh2) demonstrates depletion of the pro-form and catalytically active form of MMP-2. Amido black stain (AB) of the membrane shows equivalent loading between samples. **d** Western analysis of chromatin as described above. **e** Growth curve of expanding U2OS pLKO.1 negative control and two different pLKO.1-MMP-2 shRNAs (sh1 and sh2). The total number of cells at each experimental time point was determined (y-axis) at 24-hour intervals (x-axis), with day 0 representing the point of confluency, as illustrated in Fig. 1a.

**Additional file 2: Figure S2.** **a** Normalized read counts of MMP-2 transcripts (y-axis) from three independent biological replicate RNA-seq experiments in the U2OS subconfluent (-2 days, red), confluent (0 days, green) and over-confluent (+2 days, blue) cells (x-axis). **b** Normalized read counts of MMP-2 transcripts (y-axis) from three independent biological replicate RNA-seq experiments in the over-confluent (+2) stable U2OS pLKO.1 negative control or pLKO.1-MMP-2 shRNA cells (sh1 and sh2) cell lines. **c** Principal component analysis comparing the three independent biological replicate RNA-seq experiments in the over-confluent (+2) stable U2OS pLKO.1 negative control (red), pLKO.1-MMP2sh1 (green) and pLKO.1-MMP2sh2 (blue) cell lines. PC1 (x-axis) is plotted versus PC2 (y-axis) with the variance percentage indicated. **d** Volcano plots generated from RNA-Seq data of U2OS pLKO.1 control versus MMP2sh2 subconfluent (-2 days, left), confluent (0 days, middle) and over-confluent (+2 days, right) cells. The log adjusted fold change in expression (x-axis) was plotted relative to an adjusted p-value cutoff of 0.05 (y-axis). The number of significantly downregulated (red) and upregulated (blue) genes in the MMP2sh2 cells are indicated.

**Additional file 3: Figure S3.** **a** Illustration of ProMMP2-3xHA. The full length MMP-2 cDNA was cloned into a custom p.Lenti vector containing three sequential HA tags at the C-terminus (top). The different domains of MMP-2 are indicated: propeptide (Pro), enzymatic (Enz), fibronectin type-II repeats (FN), collagenase-like 2 (Col) and four hemopexin repeats (Hex) (bottom). **b** Western analysis of purified cytoplasmic extracts from 293T negative control cells, C2C12 myoblasts (MB) and differentiated myotubes (MT), and U2OS +2 wild type (WT) and ProMMP2-3xHA cells using an MMP-2 antibody. ProMMP2-3xHA and the endogenous MMP-2 pro-form and catalytically active form are indicated. **c** Purified U2OS over-confluent (+2) ProMMP2-3xHA nuclei were treated with MNase for 4 minutes prior to nuclear fractionation to isolate soluble euchromatin (S1), heterochromatin (S2) or insoluble chromatin (P). Western analysis of each fraction using an HA antibody was performed to detect ProMMP2-3xHA, as indicated (right). Amido black staining of the blot showing relative protein abundance between samples. **d** The S1 and S2 fractions from U2OS +2 wild type negative control cells (-) and U2OS +2 ProMMP2-3xHA cells (+) were pooled for ChIP using either a rabbit IgG negative control or HA antibody. Western analysis was performed on the ChIP-eluted samples using an HA antibody. The ProMMP2-3xHA and IgG heavy chain are indicated. **e** Optimization of native ChIP (nChIP). Nuclei were incubated with MNase (1 unit per million nuclei) for the indicated times (left). The resulting soluble S1 and S2 chromatin fractions were pooled, DNA was purified and fractionated by agarose electrophoresis. The mono- and di-nucleosomal bands are indicated. DNA from the 4’ MNase digest was analyzed using an Agilent Bioanalyzer High Sensitivity DNA Assay (right). Fluorescence units (y-axis) are plotted to base pairs (x-axis). The 4’ MNase digest was optimal, yielding > 90% mono- and di-nucleosomes, and was used for all ChIP experiments in this study. **f** Individual plot profiles (top) and heatmaps (bottom) of ProMMP2-3xHA and wild type (WT) negative control HA-nChIP-Seq replicates. RPKM normalized signal is plotted for each, centered over the 7,216 ProMMP2-3xHA called peaks and extending +/- 4 kb. **g** Venn diagram showing overlap between the individual ProMMP2-3xHA called peaks from each of the three independent HA-nChIP-Seq biological replicates. **h** Pearson correlation comparing the ProMMP2-3xHA called peaks between the three independent HA-nChIP-Seq biological replicates.

**Additional file 4: Figure S4.** **a** Venn diagram showing overlap of ProMMP2-3xHA peaks within 1 kb of all DNA accessible regions (ATAC-seq) throughout the genome. **b** Representative gene browser image of the POR gene and HOXA gene cluster (**c**). Distribution of ProMMP2-3xHA, RNA Pol II, ATAC-Seq and indicated H3 modification enrichments across the loci are displayed relative to the RPKM signal of each (y-axis). The ProMMP2-3xHA called peaks and TSSs are indicated (bottom).

**Additional file 5: Figure S5.** **a** Heatmaps and average peak profiles of ProMMP2-3xHA, RNA PolII, ATAC-Seq, H3K4me3 and H3K18ac enrichment centered at TSSs over a +/- 2 kb window (x-axis) versus average RPKM intensity (y-axis) for the 672 differentially expressed genes in MMP-2 depleted U2OS cells (Fig. 2c). The number of genes downregulated (top) or upregulated (bottom) following MMP-2 depletion are indicated. **b** Heatmaps and average peak profiles of ProMMP2-3xHA, RNA PolII, ATAC-Seq, H3K4me3 and H3K18ac enrichment centered at TSSs over a +/- 2 kb window (x-axis) versus average RPKM intensity (y-axis). Data was clustered into three groups: Protein coding TSSs with both ProMMP2-3xHA and H3K4me3 called peaks (top), those with H3K4me3 but no ProMMP2-3xHA called peaks (middle) and those lacking any called peaks (bottom). **c** Venn diagram showing overlap of TSSs with ProMMP2-3xHA called peaks (blue) and H3K4me3 called peaks genome wide. **d** Boxplot showing the average expression (transcripts per million) of all genes in each group from **S5b**. Median expression of each group is indicated by the black bar with error bars denoting standard deviation. Upper and lower quartiles are represented by the boxes. **** represents a p-value of <1e-100, calculated using the Wilcoxon rank sum test.

**Additional file 6: Figure S6.** **a** The average ChIP-Seq signal intensities (y-axis) across the entire gene bodies of the 5,889 ProMMP2-3xHA narrow peak genes (red) and 870 broad peak genes (blue), centered at transcription start site (TSS). Smaller genes were scaled up and longer genes scaled down to achieve an equivalent comparison between TSS and transcription end site (TES), with the distance between TSS/TES set to 5 kb. Signal plotted +/- 2 kb (x-axis) of TSS or TES versus average RPKM intensity (y-axis). (bottom) The average ChIP-Seq signal intensities (y-axis) of elongating RNA PolII (PolII-S2P) across the gene bodies of the ProMMP2-3xHA narrow peak genes (red) and broad peak genes (blue), as described in a. **b** IGV genome browser image of the SOX9 gene that contains a sharp ProMMP2-3xHA peak at TSS and a broad region that extends from the TSS peak through the gene body (top tract). Other ChIP-Seq enrichment tracts for RNA PolII, DNA accessible regions (ATAC) and indicated histone H3 modifications are plotted relative to the RPKM signal of each (y-axis). The protein coding (PC) TSSs and the ProMMP2-3xHA called peaks are indicated (bottom). **c** Boxplot comparing the average gene length (y-axis) of all canonical protein coding genes (yellow) to the average length of the ProMMP2-3xHA narrow peak genes (red) and broad peak genes (blue). Black bar indicates the median with error bars denoting standard deviation. Upper and lower quartiles are represented by the boxes. **** represents a p-value of <1e-100 calculated using a Wilcoxon rank sum test. Gene length values were calculated using ENSEMBL canonical protein coding gene start and end sites. **d** Heatmaps and average peak profiles of ProMMP2-3xHA, RNA PolII, RNA Pol II Ser2P, ATAC-Seq, H3K4me3 and H3K18ac signal centered over regions in a +/- 5 kb window (x-axis) versus average RPKM intensity (y-axis). ProMMP2-3xHA peaks were clustered based on peak size: 0-2 kb, 2-5kb, 5-10 kb and >10 kb, as indicated. Right: expanded heatmaps and average peak profiles of the ProMMP2-3xHA regions spanning 5-10 kb and >10 kb. **e** Heatmaps and average peak profiles of ProMMP2-3xHA, RNA PolII, RNA PolII Ser2P, ATAC-Seq, H3K4me3 and H3K18ac signal centered at TSS in a -5/+10 kb window (x-axis) versus average RPKM intensity (y-axis). Genes were clustered based on broad (top) or narrow (bottom) ProMMP2-3xHA peaks.

**Additional file 7: Figure S7.** **a** Normalized read counts of CTSB transcripts (y-axis) from three independent biological replicate RNA-seq experiments in the U2OS subconfluent (-2 days, blue), confluent (0 days, green) and over-confluent (+2 days, red) cells (x-axis). **b** RT-qPCR analysis of total RNA purified from cells stably transduced with either a pLKO.1 control or two different pLKO.1-CSTB shRNAs (sh1 and sh2). CTSB expression was normalized to GAPDH control and plotted relative to the pLKO.1 control (y-axis). Three independent biological replicates were performed to generate standard deviation (error bars). **c** Procleave prediction software was queried using the Histone H3.1 substrate (aa 1-50) with either the MMP-2 (left) or CTSB (right) proteases. Blue represents a significant score (>0.5) and red indicates a lower significance score (<0.5). Position indicates the starting amino acid of the remaining H3cl product. **d** Illustration of the in silico predicted H3NT proteolysis sites of MMP-2 and CTSB with the actual H3cl products observed by Western analysis of chromatin purified from 293T negative control, C2C12 myotube MMP-2 generated H3∆18 positive control and U2OS +2 (over-confluent) or -2 (proliferating) cells. The predicted molecular weight of each H3cl product is indicated.

**Additional file 8: Figure S8.** **a** Heatmaps and average peak profiles of ProMMP2-3xHA, RNA PolII, ATAC-Seq, H3K4me3 and H3K18ac signal centered at TSSs over a +/- 2 kb window (x-axis) versus average RPKM intensity (y-axis). Signal was plotted over three groups: TSSs with ProMMP2-3xHA and H3K4me3 called peaks (top), those with H3K4me3 called peaks but no ProMMP2-3xHA called peaks (middle), and those with H3K18ac called peaks but no H3K4me3 (bottom). The number of genes in each group is indicated. **b** Average Ct values (y-axis) of the H3 control ChIP-qPCR performed in U2OS wild type (WT, yellow) and MMP-2 depleted cells (MMP2sh1, salmon) are plotted (y-axis) for the indicated ProMMP2-3xHA positive gene loci and a negative control locus (gene desert) (x-axis). The average and standard deviation between three independent biological replicates are shown.

**Additional file 9: Table S1.** Genes downregulated across all 3 stages of U2OS cell growth. **Table S2.** Genes upregulated across all 3 stages of U2OS cell growth. **Table S3.** ProMMP2-3xHA bound transcription start sites. **Table S4.** Genes with narrow ProMMP2-3xHA peaks at the transcription start site. **Table S5.** Genes with broad ProMMP2-3xHA regions going into the gene body. **Table S6.** Antibodies and their dilutions/concentrations. **Table S7.** RT-qPCR Primers. **Table S8.** ChIP-qPCR Primers. **Table S9.** Publicly available genomic datasets downloaded for analysis.

**Additional file 10.** ChIP and ATAC-seq Data Analysis, RNA-seq Data Analysis.

## Data Availability

All ChIP-seq and RNA-seq data have been submitted to the GEO archive under the superseries GSE224887 (Subseries GSE224885 and GSE224886). All data and code is available upon further request from the authors.

## Abbreviations

H3NT: Histone H3 N-terminal tail
H3cl: Histone H3 cleaved product
PTM: Post-translational modification
MMP-2: Matrix metalloproteinase 2
TSS: Transcription start site
CTSB: Cathepsin B
NT: N-terminal tail
CTSL: Cathepsin L
MMP-9: Matrix metalloproteinase 9
nChIP: Native chromatin immunoprecipitation
MB: Myoblasts
MT: Myotubes
ECM: Extracellular matrix
Cyto: Cytosolic extracts
NE: Nuclear extracts
Chr: Insoluble chromatin
WT: Wild type
ATAC: Assay for Transposase-accessible chromatin
Seq: Sequencing
MNase: Micrococcal nuclease
PC: Protein coding
TPM: Transcripts per million
ShRNA: Short hairpin RNA
AB: Amido black
RT-qPCR: Reverse transcriptase, quantitative polymerase chain reaction
ChIP-qPCR: Chromatin immunoprecipitation, quantitative polymerase chain reaction
GO: Gene ontology
Pro: Proform
S1: Soluble fraction 1
S2: Soluble fraction 2
P: Pellet
RNA-PolII: RNA polymerase II
Nuc: Nucleosome
Ser2p: RNA polymerase II C-terminal domain serine 2 phosphorylation
chr: Chromosome
Kb: Kilobases
bp: Base pair
kDa: Kilodalton
H3.cs1: H3 cut site 1
WB: Western blot
Ct: Cycle threshold
UTR: Untranslated region
ncRNA: Non-coding RNA
TTS: Transcription termination site
