# Supplementary figures and images for: The MMP-2 histone H3 N-terminal tail protease is selectively targeted to the transcription start sites of active genes
Source: Epigenetics Chromatin. 2023 May 10;16:16. doi: 10.1186/s13072-023-00491-w (PMC10170761; doi:10.1186/s13072-023-00491-w)

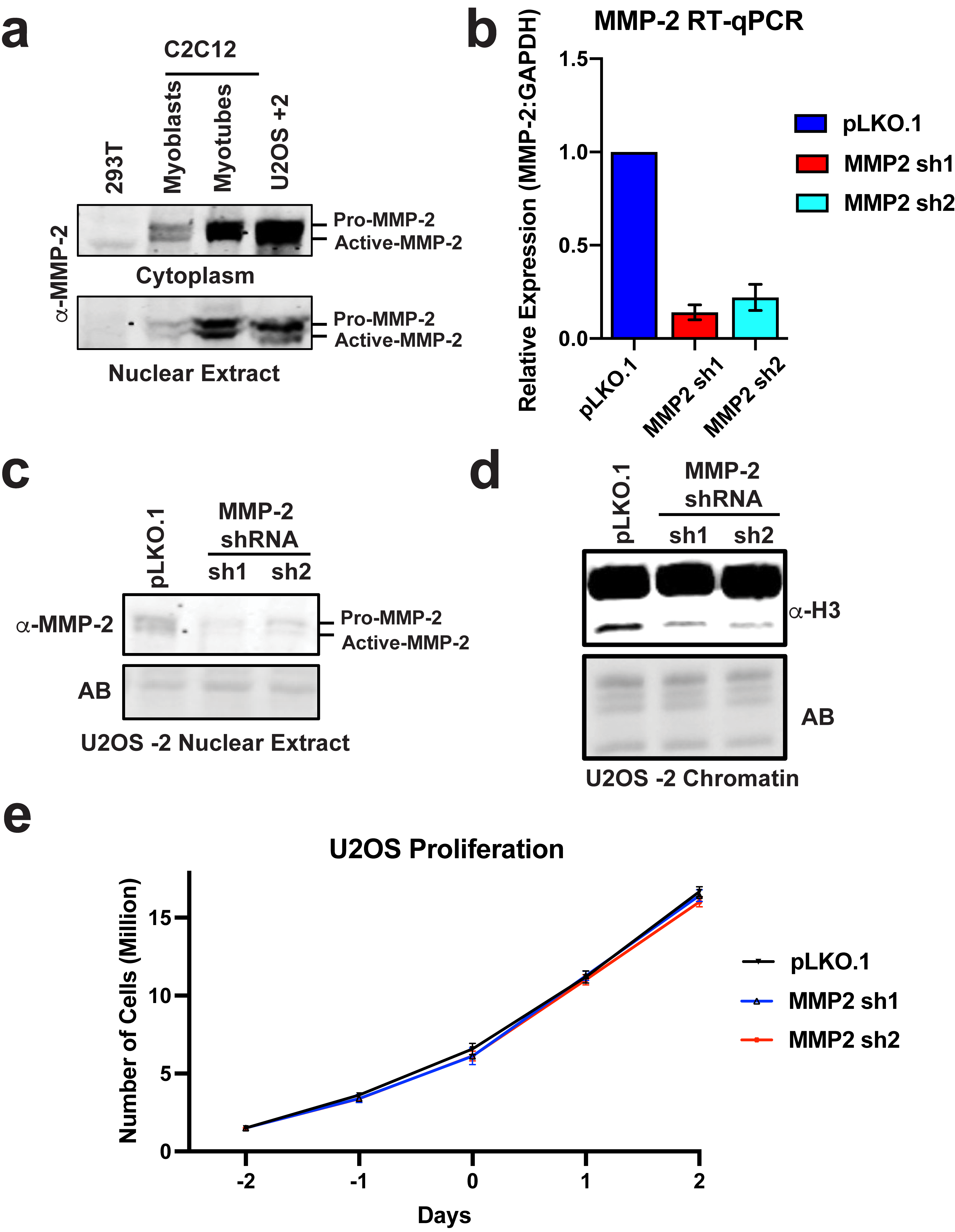

Supplement: Supplementary file 1 — Additional file 1: Figure S1. a Western analysis of cytoplasmic and nuclear extracts purified from 293T negative control cells, C2C12 myoblast and myotube positive control, and U2OS +2 cells. An MMP-2 antibody detects the pro-form and catalytically active forms of MMP-2, as indicated. b RT-qPCR analysis of total RNA purified from cells stably transduced with either a pLKO.1 control or two different pLKO.1-MMP-2 shRNAs (sh1 and sh2). MMP-2 expression was normalized to GAPDH control and plotted relative to the pLKO.1 control (y-axis). Three independent biological replicates were performed to generate standard deviation (error bars). c Western analysis of nuclear extracts purified from subconfluent (-2) U2OS pLKO.1 negative control or two different pLKO.1-MMP-2 shRNAs (sh1 and sh2) demonstrates depletion of the pro-form and catalytically active form of MMP-2. Amido black stain (AB) of the membrane shows equivalent loading between samples. d Western analysis of chromatin as described above. e Growth curve of expanding U2OS pLKO.1 negative control and two different pLKO.1-MMP-2 shRNAs (sh1 and sh2). The total number of cells at each experimental time point was determined (y-axis) at 24-hour intervals (x-axis), with day 0 representing the point of confluency, as illustrated in Fig. 1a. [file 13072_2023_491_MOESM1_ESM.png]

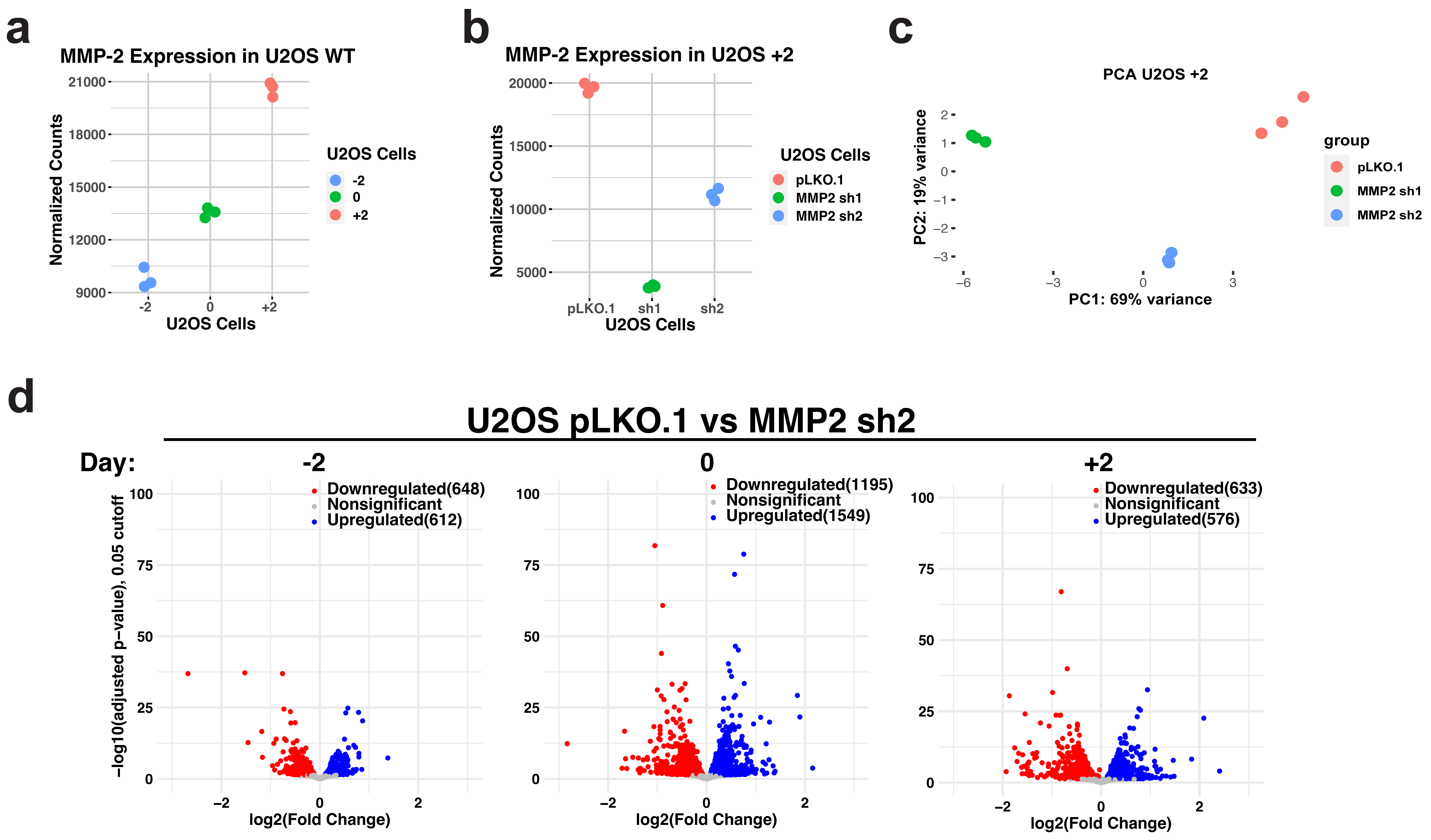

Supplement: Supplementary file 2 — Additional file 2: Figure S2. a Normalized read counts of MMP-2 transcripts (y-axis) from three independent biological replicate RNA-seq experiments in the U2OS subconfluent (-2 days, red), confluent (0 days, green) and over-confluent (+2 days, blue) cells (x-axis). b Normalized read counts of MMP-2 transcripts (y-axis) from three independent biological replicate RNA-seq experiments in the over-confluent (+2) stable U2OS pLKO.1 negative control or pLKO.1-MMP-2 shRNA cells (sh1 and sh2) cell lines. c Principal component analysis comparing the three independent biological replicate RNA-seq experiments in the over-confluent (+2) stable U2OS pLKO.1 negative control (red), pLKO.1-MMP2sh1 (green) and pLKO.1-MMP2sh2 (blue) cell lines. PC1 (x-axis) is plotted versus PC2 (y-axis) with the variance percentage indicated. d Volcano plots generated from RNA-Seq data of U2OS pLKO.1 control versus MMP2sh2 subconfluent (-2 days, left), confluent (0 days, middle) and over-confluent (+2 days, right) cells. The log adjusted fold change in expression (x-axis) was plotted relative to an adjusted p-value cutoff of 0.05 (y-axis). The number of significantly downregulated (red) and upregulated (blue) genes in the MMP2sh2 cells are indicated. [file 13072_2023_491_MOESM2_ESM.png]

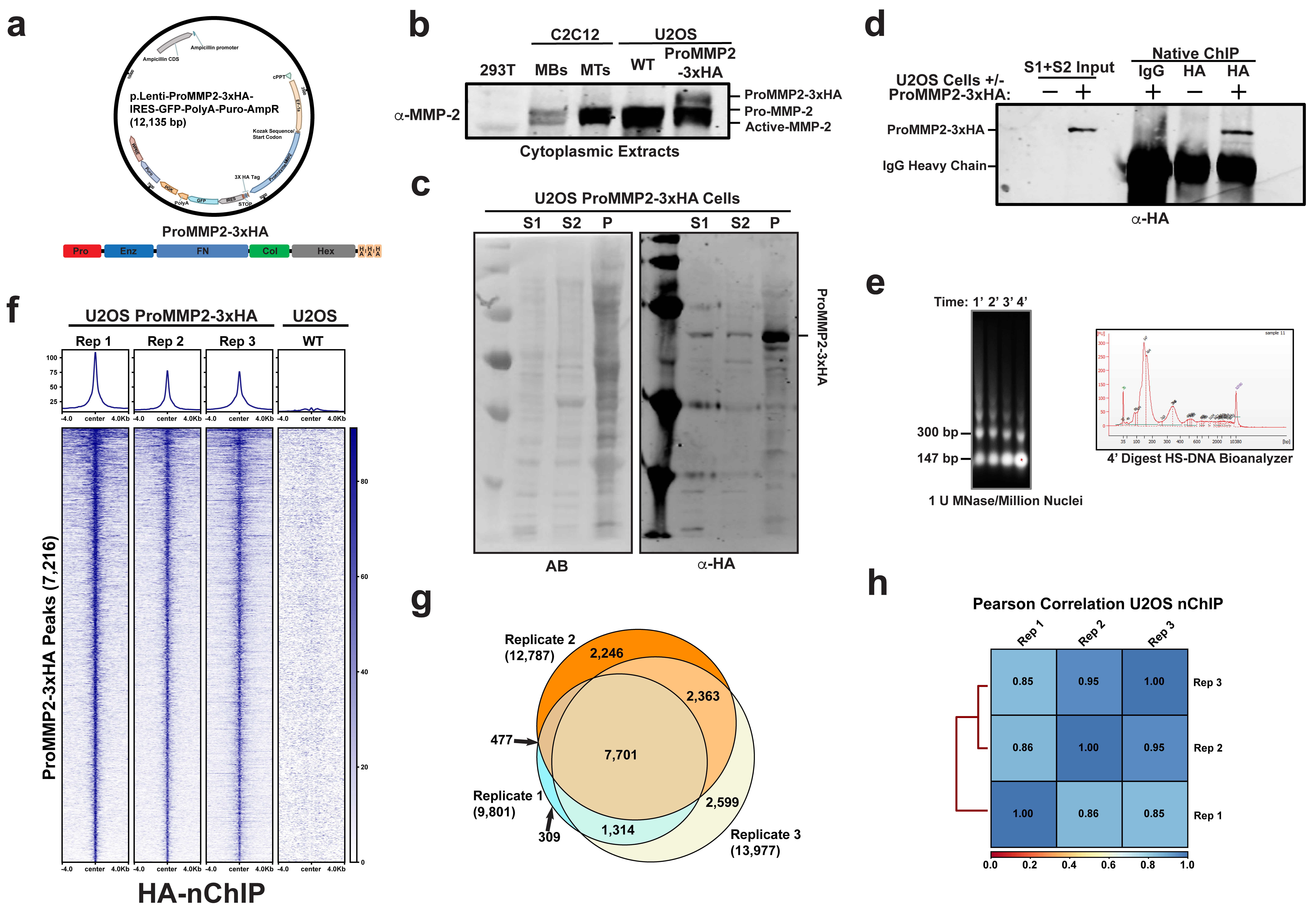

Supplement: Supplementary file 3 — Additional file 3: Figure S3. a Illustration of ProMMP2-3xHA. The full length MMP-2 cDNA was cloned into a custom p.Lenti vector containing three sequential HA tags at the C-terminus (top). The different domains of MMP-2 are indicated: propeptide (Pro), enzymatic (Enz), fibronectin type-II repeats (FN), collagenase-like 2 (Col) and four hemopexin repeats (Hex) (bottom). b Western analysis of purified cytoplasmic extracts from 293T negative control cells, C2C12 myoblasts (MB) and differentiated myotubes (MT), and U2OS +2 wild type (WT) and ProMMP2-3xHA cells using an MMP-2 antibody. ProMMP2-3xHA and the endogenous MMP-2 pro-form and catalytically active form are indicated. c Purified U2OS over-confluent (+2) ProMMP2-3xHA nuclei were treated with MNase for 4 minutes prior to nuclear fractionation to isolate soluble euchromatin (S1), heterochromatin (S2) or insoluble chromatin (P). Western analysis of each fraction using an HA antibody was performed to detect ProMMP2-3xHA, as indicated (right). Amido black staining of the blot showing relative protein abundance between samples. d The S1 and S2 fractions from U2OS +2 wild type negative control cells (-) and U2OS +2 ProMMP2-3xHA cells (+) were pooled for ChIP using either a rabbit IgG negative control or HA antibody. Western analysis was performed on the ChIP-eluted samples using an HA antibody. The ProMMP2-3xHA and IgG heavy chain are indicated. e Optimization of native ChIP (nChIP). Nuclei were incubated with MNase (1 unit per million nuclei) for the indicated times (left). The resulting soluble S1 and S2 chromatin fractions were pooled, DNA was purified and fractionated by agarose electrophoresis. The mono- and di-nucleosomal bands are indicated. DNA from the 4’ MNase digest was analyzed using an Agilent Bioanalyzer High Sensitivity DNA Assay (right). Fluorescence units (y-axis) are plotted to base pairs (x-axis). The 4’ MNase digest was optimal, yielding > 90% mono- and di-nucleosomes, and was used for all ChIP exper [file 13072_2023_491_MOESM3_ESM.png]

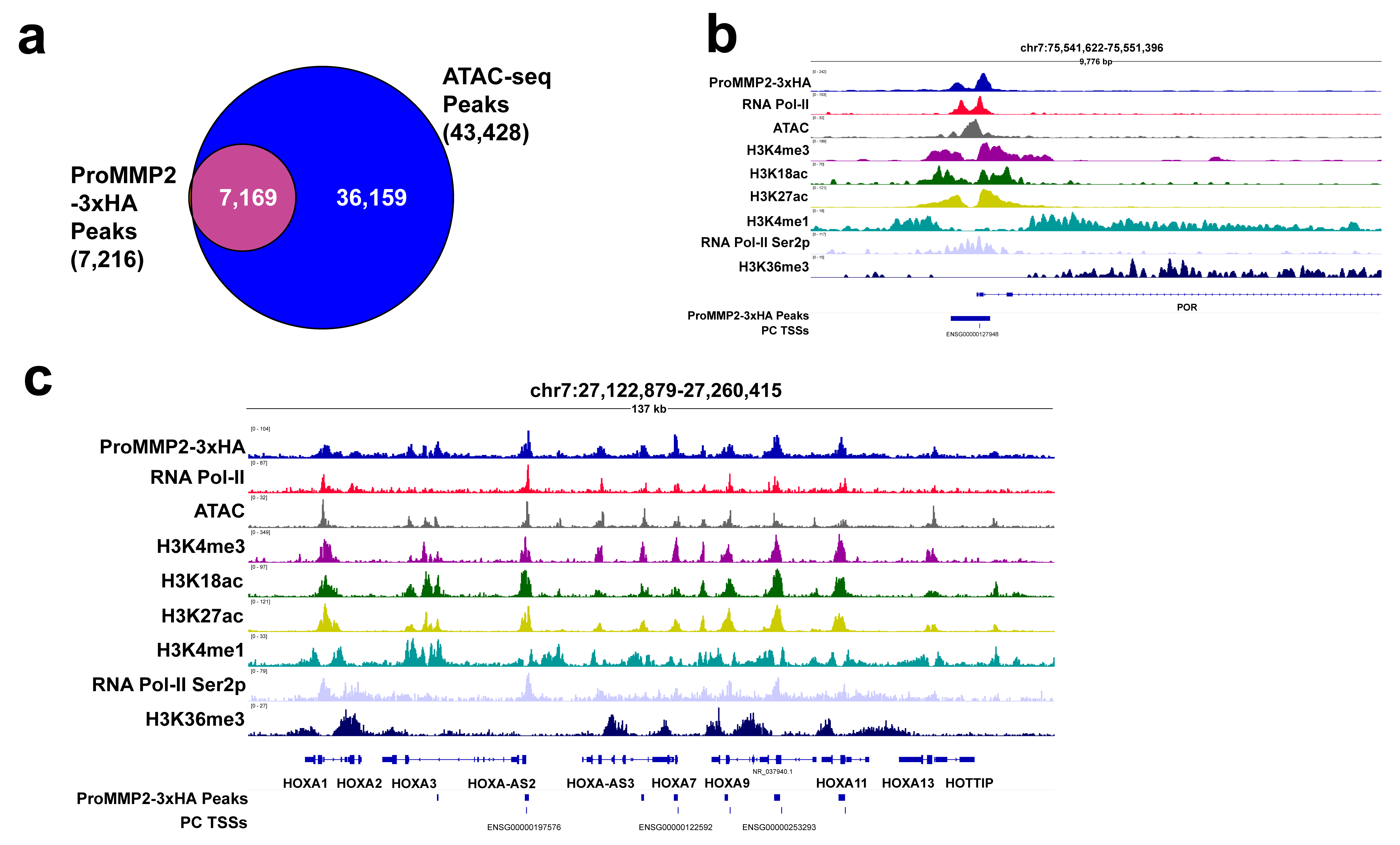

Supplement: Supplementary file 4 — Additional file 4: Figure S4. a Venn diagram showing overlap of ProMMP2-3xHA peaks within 1 kb of all DNA accessible regions (ATAC-seq) throughout the genome. b Representative gene browser image of the POR gene and HOXA gene cluster (c). Distribution of ProMMP2-3xHA, RNA Pol II, ATAC-Seq and indicated H3 modification enrichments across the loci are displayed relative to the RPKM signal of each (y-axis). The ProMMP2-3xHA called peaks and TSSs are indicated (bottom). [file 13072_2023_491_MOESM4_ESM.png]

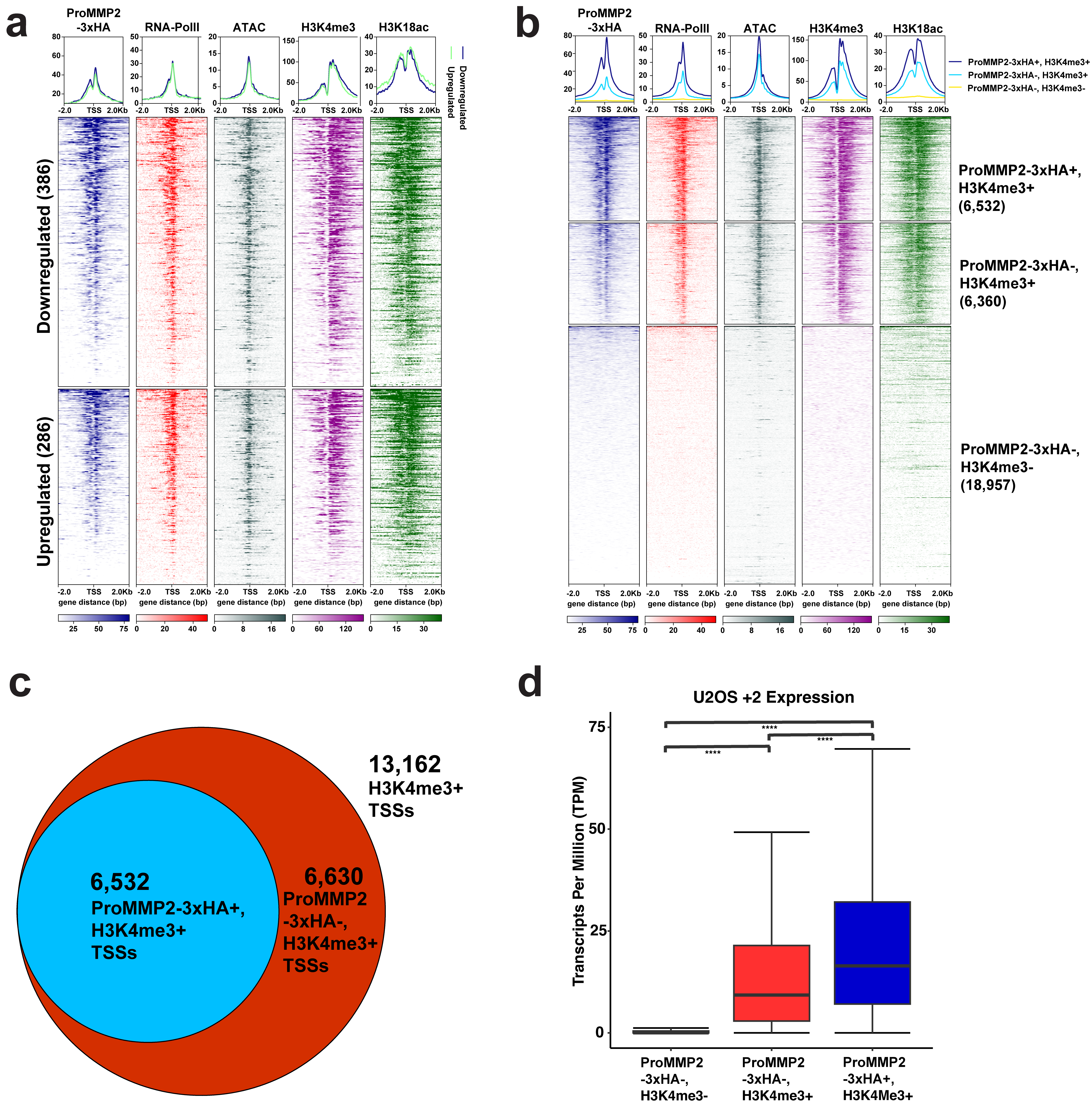

Supplement: Supplementary file 5 — Additional file 5: Figure S5. a Heatmaps and average peak profiles of ProMMP2-3xHA, RNA PolII, ATAC-Seq, H3K4me3 and H3K18ac enrichment centered at TSSs over a +/- 2 kb window (x-axis) versus average RPKM intensity (y-axis) for the 672 differentially expressed genes in MMP-2 depleted U2OS cells (Fig. 2c). The number of genes downregulated (top) or upregulated (bottom) following MMP-2 depletion are indicated. b Heatmaps and average peak profiles of ProMMP2-3xHA, RNA PolII, ATAC-Seq, H3K4me3 and H3K18ac enrichment centered at TSSs over a +/- 2 kb window (x-axis) versus average RPKM intensity (y-axis). Data was clustered into three groups: Protein coding TSSs with both ProMMP2-3xHA and H3K4me3 called peaks (top), those with H3K4me3 but no ProMMP2-3xHA called peaks (middle) and those lacking any called peaks (bottom). c Venn diagram showing overlap of TSSs with ProMMP2-3xHA called peaks (blue) and H3K4me3 called peaks genome wide. d Boxplot showing the average expression (transcripts per million) of all genes in each group from S5b. Median expression of each group is indicated by the black bar with error bars denoting standard deviation. Upper and lower quartiles are represented by the boxes. **** represents a p-value of <1e-100, calculated using the Wilcoxon rank sum test. [file 13072_2023_491_MOESM5_ESM.png]

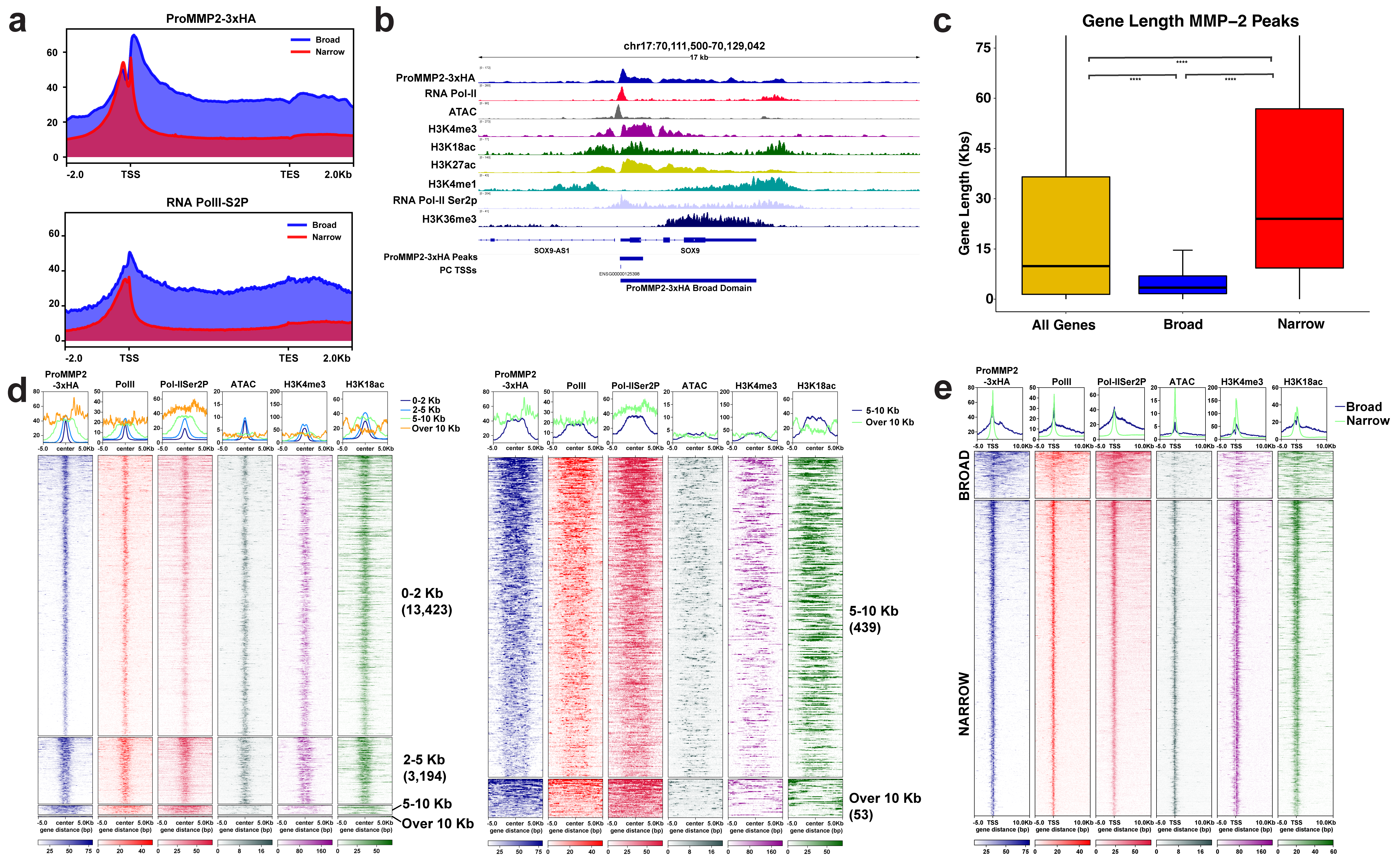

Supplement: Supplementary file 6 — Additional file 6: Figure S6. a The average ChIP-Seq signal intensities (y-axis) across the entire gene bodies of the 5,889 ProMMP2-3xHA narrow peak genes (red) and 870 broad peak genes (blue), centered at transcription start site (TSS). Smaller genes were scaled up and longer genes scaled down to achieve an equivalent comparison between TSS and transcription end site (TES), with the distance between TSS/TES set to 5 kb. Signal plotted +/- 2 kb (x-axis) of TSS or TES versus average RPKM intensity (y-axis). (bottom) The average ChIP-Seq signal intensities (y-axis) of elongating RNA PolII (PolII-S2P) across the gene bodies of the ProMMP2-3xHA narrow peak genes (red) and broad peak genes (blue), as described in a. b IGV genome browser image of the SOX9 gene that contains a sharp ProMMP2-3xHA peak at TSS and a broad region that extends from the TSS peak through the gene body (top tract). Other ChIP-Seq enrichment tracts for RNA PolII, DNA accessible regions (ATAC) and indicated histone H3 modifications are plotted relative to the RPKM signal of each (y-axis). The protein coding (PC) TSSs and the ProMMP2-3xHA called peaks are indicated (bottom). c Boxplot comparing the average gene length (y-axis) of all canonical protein coding genes (yellow) to the average length of the ProMMP2-3xHA narrow peak genes (red) and broad peak genes (blue). Black bar indicates the median with error bars denoting standard deviation. Upper and lower quartiles are represented by the boxes. **** represents a p-value of <1e-100 calculated using a Wilcoxon rank sum test. Gene length values were calculated using ENSEMBL canonical protein coding gene start and end sites. d Heatmaps and average peak profiles of ProMMP2-3xHA, RNA PolII, RNA Pol II Ser2P, ATAC-Seq, H3K4me3 and H3K18ac signal centered over regions in a +/- 5 kb window (x-axis) versus average RPKM intensity (y-axis). ProMMP2-3xHA peaks were clustered based on peak size: 0-2 kb, 2-5kb, 5-10 kb and >10 kb, as indicated. Right: expanded hea [file 13072_2023_491_MOESM6_ESM.png]

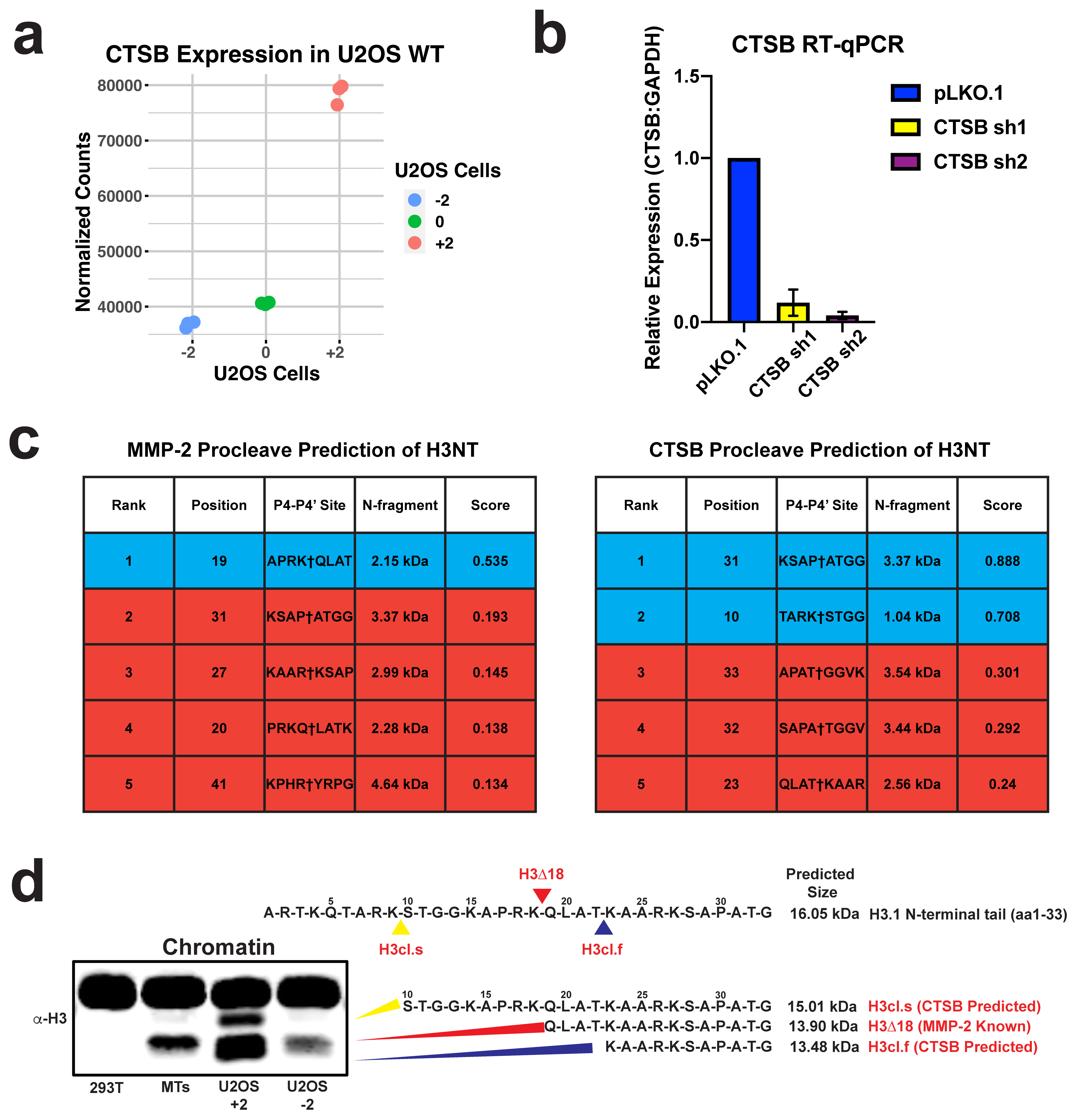

Supplement: Supplementary file 7 — Additional file 7: Figure S7. a Normalized read counts of CTSB transcripts (y-axis) from three independent biological replicate RNA-seq experiments in the U2OS subconfluent (-2 days, blue), confluent (0 days, green) and over-confluent (+2 days, red) cells (x-axis). b RT-qPCR analysis of total RNA purified from cells stably transduced with either a pLKO.1 control or two different pLKO.1-CSTB shRNAs (sh1 and sh2). CTSB expression was normalized to GAPDH control and plotted relative to the pLKO.1 control (y-axis). Three independent biological replicates were performed to generate standard deviation (error bars). c Procleave prediction software was queried using the Histone H3.1 substrate (aa 1-50) with either the MMP-2 (left) or CTSB (right) proteases. Blue represents a significant score (>0.5) and red indicates a lower significance score (<0.5). Position indicates the starting amino acid of the remaining H3cl product. d Illustration of the in silico predicted H3NT proteolysis sites of MMP-2 and CTSB with the actual H3cl products observed by Western analysis of chromatin purified from 293T negative control, C2C12 myotube MMP-2 generated H3∆18 positive control and U2OS +2 (over-confluent) or -2 (proliferating) cells. The predicted molecular weight of each H3cl product is indicated. [file 13072_2023_491_MOESM7_ESM.png]

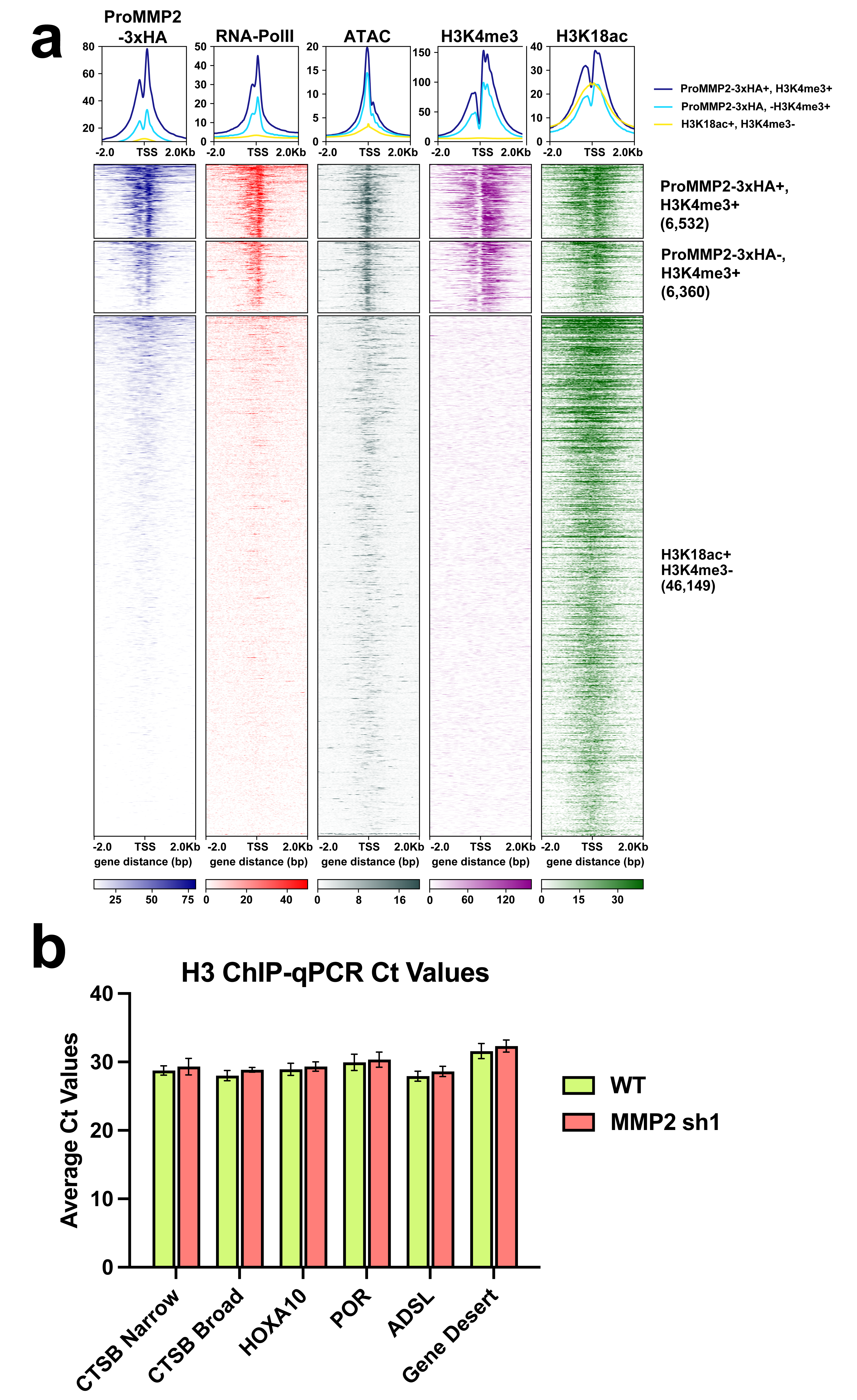

Supplement: Supplementary file 8 — Additional file 8: Figure S8. a Heatmaps and average peak profiles of ProMMP2-3xHA, RNA PolII, ATAC-Seq, H3K4me3 and H3K18ac signal centered at TSSs over a +/- 2 kb window (x-axis) versus average RPKM intensity (y-axis). Signal was plotted over three groups: TSSs with ProMMP2-3xHA and H3K4me3 called peaks (top), those with H3K4me3 called peaks but no ProMMP2-3xHA called peaks (middle), and those with H3K18ac called peaks but no H3K4me3 (bottom). The number of genes in each group is indicated. b Average Ct values (y-axis) of the H3 control ChIP-qPCR performed in U2OS wild type (WT, yellow) and MMP-2 depleted cells (MMP2sh1, salmon) are plotted (y-axis) for the indicated ProMMP2-3xHA positive gene loci and a negative control locus (gene desert) (x-axis). The average and standard deviation between three independent biological replicates are shown. [file 13072_2023_491_MOESM8_ESM.png]
