# Supplementary material for: The MMP-2 histone H3 N-terminal tail protease is selectively targeted to the transcription start sites of active genes
Source: Epigenetics Chromatin. 2023 May 10;16:16. doi: 10.1186/s13072-023-00491-w (PMC10170761; doi:10.1186/s13072-023-00491-w)
Supplement: Supplementary file 10 — Additional file 10. ChIP and ATAC-seq Data Analysis, RNA-seq Data Analysis. [file 13072_2023_491_MOESM10_ESM.docx]

**SUPPLEMENTAL METHODS**

**ChIP and ATAC-seq Data Analysis**

The quality of raw reads was estimated with FastQC (Illumina, v0.11.9). All published ChIP-seq and ATAC-seq data (listed in supplemental table 9) were downloaded from GEO ( ) and reanalyzed as subsequently described. Raw reads were trimmed to remove any adapters using TrimGalore (0.6.0). Sequenced reads were aligned to the human genome assembly GRCh37 (hg19) using bowtie2 (2.3.5.1) in paired end mode with the options –no-mixed and –no-discordant. Low quality reads were filtered out using Samtools (1.9) with a cutoff MAPQ of 30, and duplicated reads removed using the Picard toolkit (<http://broadinstitute.github.io/picard/>). Unique read files were used for peak calling using MACS2 (3.0.0a6) using the callpeaks function with the options -f BAMPE -q 0.05 –min-length 147 –nomodel, using the corresponding input or control IP as -c. Narrow and broad peak calling modes were both utilized and then merged to capture all peaks.

For visualization, each sample was normalized using RPKM and genome coverage tracks (bigwig files) were produced using deepTools (3.5.1) bamCoverage function with the options –binSize 10 –smoothLength 30 and using an ENCODE hg19 blacklist file (<https://doi.org/10.1038/s41598-019-45839-z>) to discard regions with consistent non-specific signal. High correlation between replicates was visualized using multiBamSummary (-bs 10000) and plotCorrelation (--corMethod pearson) functions from deepTools. Heatmaps were generated using computeMatrix (--binSize 10) and plotHeatmap (or plotProfile) functions from deepTools. Canonical Protein Coding TSSs list was generated in R by intersecting the UCSC hg19 knownCanonical and knownGene to get canonical protein coding genes, and TSSs defined as the gene start +/- 1bp (depending on gene strand). All heatmaps plotted centered on the TSS account for gene directionality.

­Peak overlap was performed using bedtools2 (2.27.1) window function with the options -w 1000 and either -u or -v. Venn diagrams were made using Eulerr in R (https://cran.r-project.org/web/packages/eulerr/vignettes/gallery.html). Peak annotation of ProMMP2-3xHA peaks was performed using homer (v4.11) annotatePeaks.pl function. Broad ProMMP2-3xHA regions were called by splitting the genome into 1000 bp windows (with a 100 bp sliding window) and getting the average number of reads per window using deepTools multiBigWigSummary. ProMMP2-3xHA signal per window was compared to the U2OS-HA mock IP signal as a control. Regions with signal at least 1 standard deviation above the average and two-fold signal over the U2OS-HA mock IP were deemed significant (analysis performed in R). All overlapping regions and regions within 500 bp of each other were merged using bedtools2 merge (-d 501). Genes with >25% of the gene body covered by a broad domain were computed using Bedtools intersect function (provided as supplementary bed file). All code is available upon request.

**RNA-seq Data Analysis**

The quality of raw reads was estimated with FastQC (Illumina, v0.11.9, <http://www.bioinformatics.bbsrc.ac.uk/projects/fastqc>). Raw reads were trimmed to remove any adapters using Trim Galore (0.6.0, <https://www.bioinformatics.babraham.ac.uk/projects/trim_galore/> ). Files were aligned using STAR (2.7.3a) to the hg19 genome. Low quality reads were filtered out using Samtools as described above. HTSeq (2.0.0) was used to generate raw transcript counts using the options –minaqual=10 –stranded=no –type=exon –mode=union and the resulting files used as input for DESeq2 (<https://bioconductor.org/packages/release/bioc/html/DESeq2.html> ). Transcripts per million counts were calculated using the RSEM (1.3.3) function rsem-calculate-expression with the –paired-end option. DESeq2 was utilized as previously described (ref) with a cutoff using a padj < 0.05 unless otherwise noted. Corresponding volcano plots were generated using ggplot2(3.4.0) geom_point function. Gene ontology analysis was performed using Enrichr (<https://maayanlab.cloud/Enrichr/>). Integration of TPM counts and ChIP-seq peaks was done in R using dplyr (1.0.10) and tidyverse (1.3.2) and resulting box/violin plots performed using ggplot2. All code is available upon request.
